# Development and intra-renal delivery of renal progenitor organoids for effective integration in vivo

**DOI:** 10.1093/stcltm/szae078

**Published:** 2024-10-28

**Authors:** Diana Lim, Ickhee Kim, Qianqian Song, Ji Hyun Kim, Anthony Atala, John D Jackson, James J Yoo

**Affiliations:** Wake Forest Institute for Regenerative Medicine, Wake Forest School of Medicine, Medical Center Boulevard, Winston-Salem, NC 27157, United States; Wake Forest Institute for Regenerative Medicine, Wake Forest School of Medicine, Medical Center Boulevard, Winston-Salem, NC 27157, United States; Wake Forest Institute for Regenerative Medicine, Wake Forest School of Medicine, Medical Center Boulevard, Winston-Salem, NC 27157, United States; Wake Forest Institute for Regenerative Medicine, Wake Forest School of Medicine, Medical Center Boulevard, Winston-Salem, NC 27157, United States; Wake Forest Institute for Regenerative Medicine, Wake Forest School of Medicine, Medical Center Boulevard, Winston-Salem, NC 27157, United States; Wake Forest Institute for Regenerative Medicine, Wake Forest School of Medicine, Medical Center Boulevard, Winston-Salem, NC 27157, United States; Wake Forest Institute for Regenerative Medicine, Wake Forest School of Medicine, Medical Center Boulevard, Winston-Salem, NC 27157, United States

**Keywords:** kidney, regeneration, renal organoid, tissue engineering, clinical translation

## Abstract

Renal progenitor organoids have been proposed as a source of tissue for kidney regeneration; however, their clinical translatability has not been demonstrated due to an inability to mass-produce *comprehensive* renal progenitor organoids and the lack of an effective intra-renal delivery platform that facilitates rapid integration into functionally meaningful sites. This study addresses these shortcomings. Human-induced pluripotent stem cells were differentiated into renal progenitor cells using an established protocol and aggregated using a novel assembly method to produce high yields of organoids. Organoids were encapsulated in collagen-based scaffolds for in vitro study and in vivo implantation into mouse renal cortex. In vitro, the organoids demonstrated sustained cell viability and renal structure maturation over time. In vivo delivered organoids showed rapid integration into host renal parenchyma while showing tubular and glomerular-like structure development and maturity markers. This proof-of-concept study presents many promising results, providing a system of renal organoid formation and delivery that may support the development of clinically translatable therapies and the advancement of in vitro renal organoid studies.

## Graphical Abstract

**Graphical Abstract:**
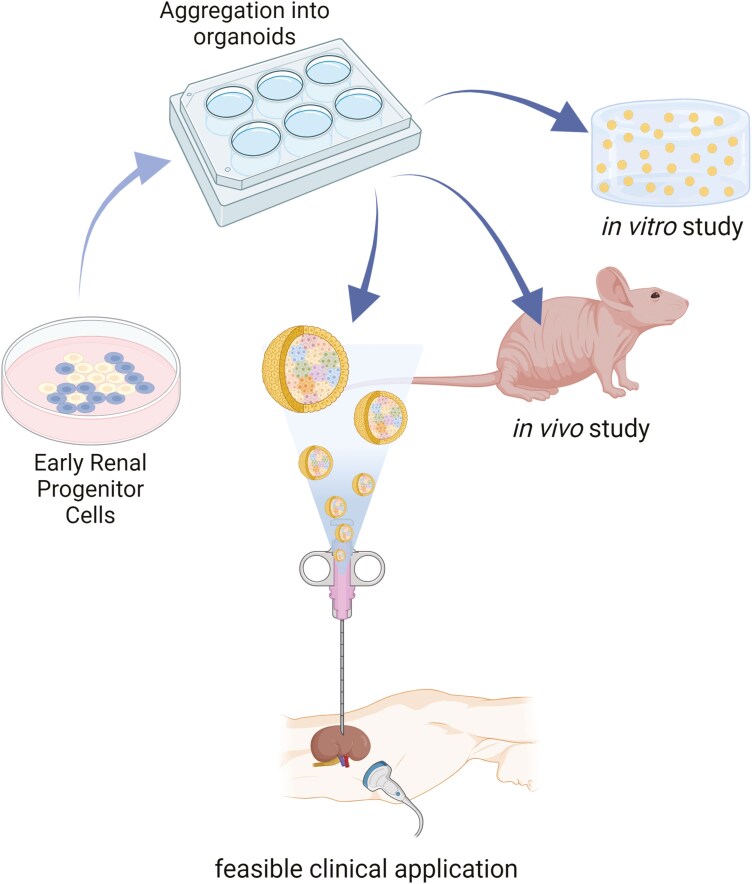

**Significance statement**

This study employs a novel system of mass-producing comprehensive renal progenitor organoids and a minimally invasive method of implantation that produces renal-like tissue in the functionally relevant site of the renal cortex. This proof-of-concept study demonstrates the ease and feasibility of taking an organoid injection-based approach to developing clinically translatable therapies for renal failure.

## Introduction

Over 50% of patients with end-stage renal disease (ESRD) do not have access to life-saving treatments of dialysis and transplantation due to a lack of transplantable organs and high maintenance care costs.^1,2^ This gap in care is predicted to grow dramatically in the next decade as the incidence of its highest causative factors, diabetes and hypertension, increases worldwide—especially in countries that lack infrastructure for current treatments.^2-5^ Studies in tissue engineering sought to eliminate donor tissue dependence by fabricating renal tissue. To this end, protocols to generate micro-tissue units called renal organoids, were introduced.^6-11^ These protocols aggregate renal cell types of differing origin or stemness to develop microarchitecture preceding in vitro study or in vivo implantation. The protocol selected determines the component of the functional kidney unit, or nephron, that is produced for study. For example, generating an organoid with early renal progenitor cells will produce more comprehensive microtissues that encompass tubular and glomerular structures. In contrast, aggregates of more mature tubule-committed cells will only generate tubule structures upon maturation.

Thus far, most renal organoid studies have been performed in vitro to study renal development and model different pathologies; while a few studies have applied renal progenitor cells and organoids to various in vivo environments, including subcutaneous, renal sub-capsular, and intrarenal environments to study and develop larger organoid-derived tissue structures.^12-16^ Subcutaneous and sub-capsular approaches are limited in their clinical applicability because tissues developed using these approaches cannot connect to the collecting system for waste removal. Additionally, many subcutaneous and subcapsular delivery findings show significant off-target cell formation—limiting the use of these implanted tissues for possible heterotopic re-implantation.^12-15,17^ Intrarenal implantation may allow for connection to the collecting duct system, rich vascularization, and signals from the renal niche that may quell the formation of off-target cells. Intrarenal organoid delivery has thus far only been demonstrated successfully by one other study that delivered tubule organoids into the parenchyma after a 5/6 nephrectomy.^18^ Though impactful, the findings of this study are limited because the 5/6 nephrectomy model changes the pressures and cues of the natural environment. Furthermore, as only tubule precursors were used in the organoid cell source, it is impossible to know if organoid-derived glomerular structures can expand and survive upon intrarenal delivery.

In developing a clinically feasible strategy for intrarenal implantation, a current limitation is the low-yield generation of more comprehensive renal progenitor organoids.^6,8,9,19^ Another limitation is that the delivery vehicle for many previous in vivo studies has been Matrigel, a solubilized basement extracellular matrix produced by mouse sarcoma cells. A more defined and controlled delivery vehicle would be favorable for clinical translation. However, the successful growth of renal progenitor organoids in such a scaffold has not been demonstrated. These limitations create a gap in knowledge about bringing established renal progenitor organoid technology closer to clinical translation in treating ESRD.

**Figure 1.**
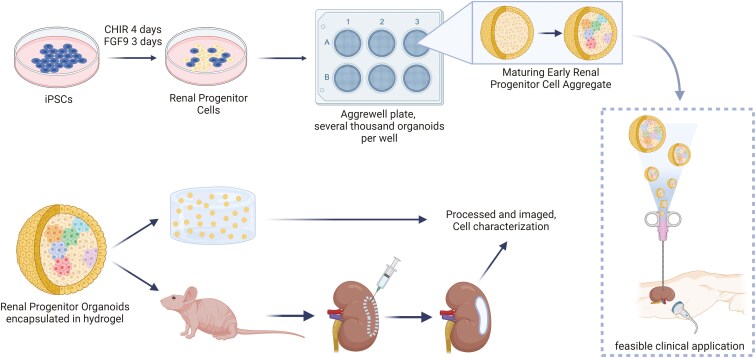
Schematic diagram of overall research strategy. Human induced pluripotent stem cells were differentiated in 2D culture using a 2-step exposure of CHIR, that induced Wnt signaling, and FGF9 to develop early renal progenitor cells (a mixture of metanephric mesenchyme (MM) and ureteric epithelium (UE) cells). The resulting progenitor cells are aggregated and matured for 5 days. The plates used in this study yielded roughly 5900 organoids per well or roughly 35 400 organoids per plate. At 5 days post-aggregation, organoids are encapsulated in a collagen-based hydrogel for in vitro culture or injection into the renal parenchyma for in vivo experiments. In vivo experiments were designed to show proof-of-concept for minimally invasive injection-based treatments (on right).

In this study, we developed a novel minimally invasive approach for intrarenal delivery of comprehensive renal progenitor organoids. We focused on modifying established renal organoid production protocols to consistently produce a high yield of organoids of uniform size, shape, and quality. We demonstrated the ease of reproducibility of the organoid formation process and evaluated the quality of organoids produced via immunohistochemistry and single-cell RNA sequencing analysis (scRNAseq). We assessed long-term cell viability and renal structure formation within a clinically feasible collagen-based delivery vehicle. Finally, we developed a strategy for the injectable application of this organoid-laden scaffold into the renal parenchyma and verified the formation and integration of organoid-derived renal structures into native kidney tissue in vivo (Figure 1). This proof-of-concept study shows promising results that provide a system of renal organoid formation and delivery that may support the development of clinically translatable therapies, as well as the advancement of in vitro renal organoid studies.

## Abbreviated methods and materials (detailed in supplemental section)

### Organoid preparation

#### Induced pluripotent stem cell maintenance

Undifferentiated Human Induced pluripotent stem cells (iPSCs) were cultured using serum-free media supplemented with ROCK inhibitor for 4-5 days.

#### iPSC differentiation in 2D culture

Induced pluripotent stem cell (IPSC) differentiation methods were adapted from those reported by Takasato et al^6^ Expanded iPSCs were collected and re-suspended in fresh serum-free APEL media with ROCK inhibitor and plated. After 1 day of incubation, media was removed and replaced with CHIR99021 supplemented medium for 4 days, followed by medium containing FGF9 and heparin for 3 days to induce differentiation into intermediate mesoderm cells. Proper differentiation was confirmed by staining for GATA3 and HOXD11.

#### Renal progenitor cell aggregation and 3D culture

Cultured cells were detached and re-suspended in CHIR99021 supplemented media for 1 hour on a non-adherent plate. Cells were re-suspended in medium containing FGF9 and heparin and distributed into wells of an Aggrewell plate at a density of 500 cells per micro-well. Cells were cultured for 5 days. Organoids were collected from the plates using a reversible strainer and re-suspended for counting. Once counted, organoids were re-suspended in the hydrogel described below.

The average diameter of organoids at different timepoints was measured by taking bright field images of 4 quadrants in each of the 6 wells within the Aggrewell plates, measuring diameters using CellSense software, and averaging the values.

The number of cells per organoid was estimated by dissociating a sample of the organoids from the same batch and counting the cells.

This cell aggregation process and following evaluation were repeated 15 times for this study.

### Cell sequencing

#### Organoid dissociation and preparation

Organoids were prepared in 2 batches. Organoids were collected, washed, enzymatically dissociated, and pipetted to break apart clusters. Debris was removed using a cell strainer and centrifugation. The collected cells were counted.

To ensure high cell viability, a dead cell removal kit was used.

#### Sequencing and analysis

Cellular suspensions were loaded on a Chromium Single Cell Controller to generate single-cell GEMs. ScRNAseq libraries were prepared. GEM-RT was performed, GEMs were broken, and the single-strand cDNA was cleaned. cDNA was amplified, cleaned, and fragmented. Indexed sequencing libraries were constructed and the barcode sequencing libraries were quantified. Sequencing libraries were loaded at 260 pM final concentration on an Illumina NovaSeq 6000 with SP-100 cycle kit, paired-end sequencing. 10x Genomics analysis platform Cell Ranger was used to process the data and generate t-SNE and UMAP renderings to identify different clusters of cells.

### Vehicle of delivery preparation and evaluation

#### Collagen hydrogel preparation

Collagen hydrogel was prepared using 0.2% rat-tail type 1 collagen with 0.25 mM of Genipin. For in vitro studies, hydrogel was mixed with and without organoids and aliquoted in 48-well plates for thermal gelation. For in vivo experiments, organoid-laden collagen gels were kept on ice until the surgical site was prepared. Genipin was added to the gel immediately before encapsulation.

#### Scanning electron microscopy (SEM) for evaluation of hydrogel degradation

Organoid-laden hydrogels and organoid-free hydrogels were cultured for 1-, 2-, and 4-week timepoints. These hydrogels were retrieved, washed, lyophilized, and critical point dried for sputter-coating and imaged. Average pore sizes were measured for each sample.

#### Assessment of cell viability in collagen-genipin hydrogel

Organoid-laden hydrogels were washed and resuspended in DMEM F:12 medium mixed with Calcein AM, Ethidium homodimer, and Hoechst stain for live/dead staining. The entire gel was imaged using confocal microscopy.

### Evaluation of organoid development

#### Sectional evaluation

Preparation of pre-encapsulation organoids for staining: Isolated organoids from day 5 post-aggregation were collected, fixed, and embedded in agarose discs prior to paraffin embedding. Preparation of hydrogel-encapsulated organoids for staining: Organoid-laden hydrogels from 1-week, 2-week, and 4-week culture timepoints were fixed in their entirety and processed for paraffin embedding.

Staining: paraffin blocks were sectioned and processed for antigen retrieval and deparaffinization followed by treatment with Triton-X and protein blocking before staining. The following antibodies were used: Sall1, Six2, GATA3, Pax2, LTL, ECAD, UMOD, WT1, NHPS1, CD31/PCAM1, PODXL, CD146/MCAM, HLA, LHX1, HOXD11, Ki67. All immunofluorescence analyses were repeated 8 times and representative images were presented. Hematoxylin and eosin (H&E) staining was also completed for each set of timepoints for morphological evaluation.

#### Confocal imaging of organoids in hydrogel

Hydrogels were fixed and blocked with protein blocking solution prior to incubation with primary and secondary antibodies. Images were taken using a confocal microscope. All analyses were repeated 7 times and representative images were presented.

#### SEM and TEM imaging of organoids

SEM imaging: done as in Section Scanning electron microscopy (SEM) for evaluation of hydrogel degradation, with imaging focusing on cell morphology.

TEM imaging: organoid-laden hydrogels were cultured for 1-, 2-, and 4-week timepoints. These hydrogels were retrieved and washed prior to fixing with glutaraldehyde in Millonig’s phosphate buffer. Samples were washed in buffer and post-fixed with osmium tetroxide in phosphate buffer. After washing, samples were dehydrated and incubated in propylene oxide in preparation for resin infiltration. Finally, the samples were gradually infiltrated with 1:1, 1:2, and pure solutions of Spurr’s resin and allowed to cure in a 70 °C oven overnight. Thin sections were obtained, stained with lead citrate and uranyl acetate, and viewed with a TEM and images captured.

#### In vivo scaffold injection

Two groups of hydrogel were prepared, a hydrogel-only control group and an organoid-laden experimental group of 2000 organoids per 50µl of hydrogel, that were kept on ice until needed for injection. The animals were divided into groups and sacrificed at 4 timepoints: 2-day, 1 week, 2 weeks, and 4 weeks (*n* = 1 normal control and *n* = 4 experimental and vehicle-only groups for each timepoint). The left kidney was injected with organoid-laden hydrogel, and the right kidney was injected with hydrogel only. 6-8 week-old male athymic mice were used. All animal procedures were performed in accordance with the NIH Guide for the Care and Use of Laboratory Animals using a protocol approved by the Institutional Animal Care and Use Committee at Wake Forest University Health Sciences (protocol number: A19-184). Animals were anesthetized with isoflurane and the left kidney was exposed via a ventral abdominal incision. A bulldog clamp was used to clamp the renal pedicle. Hydrogels were injected into the cortical region of the renal parenchyma. Once the hydrogel was ejected from the syringe, the needle was kept in place for 1 minute to prevent leakage. Following needle removal, the bulldog clamp was removed and any active bleeding was controlled, followed by wound closure.

The kidneys were retrieved, fixed, processed, and paraffin-embedded for sectioning. Staining and imaging was done as described above.

### Statistical analyses

Descriptive statistical analysis was performed to describe the variability of organoid size, organoid number, and organoid production by an expression of mean ± SE of the mean.

Analyses of scRNAseq data are discussed above in Section Sequencing and analysis.

## Results

### Scalable production of viable renal progenitor organoids

**Figure 2.**
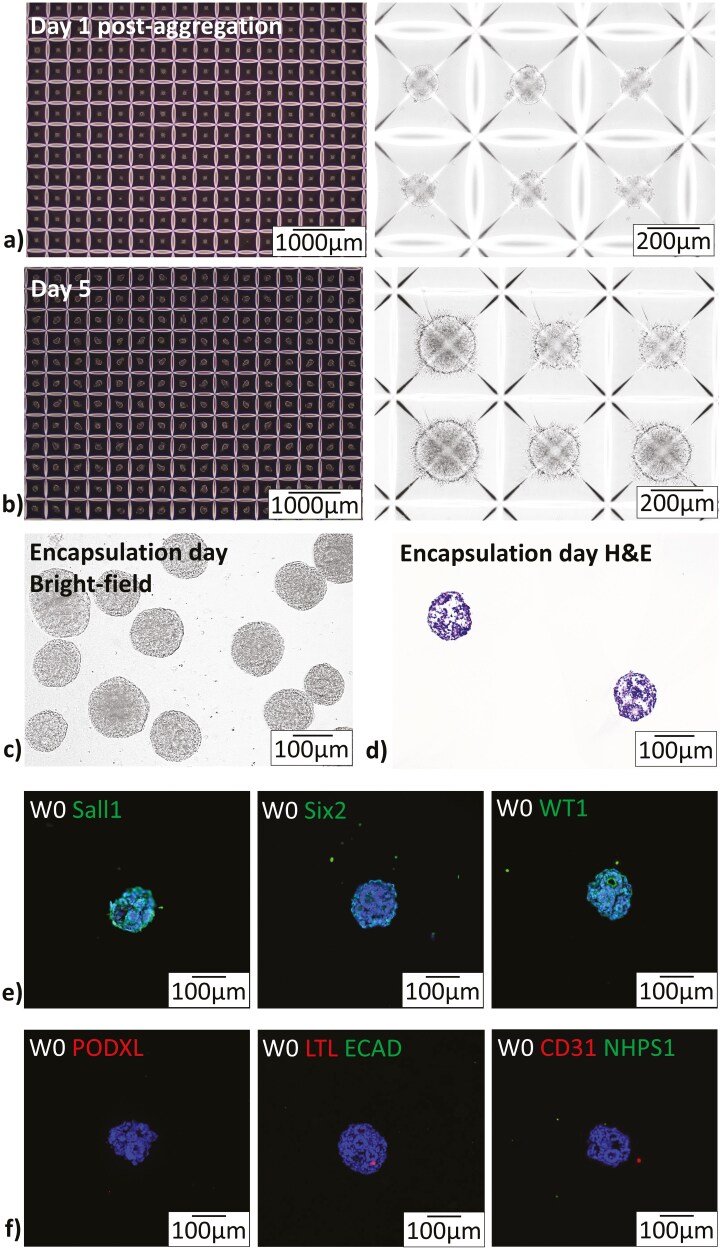
Aggregation of renal progenitor cells using an Aggrewell culture system successfully produced a high quantity of organoids with tubule-like structures and expression of early renal markers. (A) 1 day post aggregation, cohesive evenly distributed spherical organoids are seen in all micro-wells. (B) 5 days post aggregations, organoids have developed uneven surfaces and shadows indicative of internal structure formation can be seen. (C) Bright-field images of organoids encapsulated in collagen-genipin hydrogels showed more clearly the internal structures developing within organoids at day-5 post-aggregation/day 0 of encapsulation. (D) H&E images of organoids showed evidence of luminal structures with some polarity developing within the organoid at this early timepoint. (E) Positive staining for early renal markers Sall1, Six2, and WT1 in contrast to (F) little to no expression of mature renal markers PODXL, LTL, ECAD, CD31, and NHPS1. Auto-fluorescence and secondary controls are shown in Figure S4.

To reliably generate scalable yields of renal progenitor organoids with smaller diameters, we used an Aggrewell culture system. Our protocol resulted in organoids that grew from roughly 82.9 ± 6.1 µm to 193.5 ± 14.2 µm in diameter between days 1 and 5 based on bright field imaging (Figure 2a, b). Organoid yield per batch of differentiation, starting with two 10 cm plates of one million cells each, was 35 254 ± 104 organoids. Dissociation and counting of cells in organoids after 5 days in culture shows an estimated 458 ± 25 cells per organoid. These numbers were gathered from 15 × 6 well repeats as discussed in our methods.

Bright-field images showed that the micro-wells of the plate were evenly populated (Figure 2a). These images show even and smooth spherical aggregates after 1 day in culture that develop into organoids with internal structure formation after 5 days in culture (Figure 2b). H&E images clearly show structural organization (Figure 2c). For clarity, day 5 post-aggregation is the same timepoint as day 0 post-encapsulation in hydrogel.

As we used a novel method of renal progenitor cell aggregation and maturation for this organoid type, we sought to evaluate the viability and maturation of our organoids. We confirmed that the aggregated cells were early renal progenitor cells that stained positively for GATA3 indicative of ureteric epithelium (collecting duct progenitor cells), and HOXD11 indicative of metanephric mesenchyme (nephron progenitor cells) (Figure S1). After 5 days of culture, these cells organize into early tubule-like structures with luminal spaces (Figure 2d) that stain positively for early nephron markers (SALL1, Six2, and WT1) (Figure 2e) and negatively for mature markers (LTL, ECAD, CD21, and NHPS1) (Figure 2f). These features characterize organoids at the encapsulation timepoint as early nephron progenitor organoids.

### ScRNAseq identifies strong populations of renal progenitor cells

ScRNAseq was used as a higher-power method of identifying cell types within organoids at the encapsulation time-point. Our findings corroborate our immunohistochemical data and provide insight into how cells develop into renal cells within our organoids.

**Figure 3.**
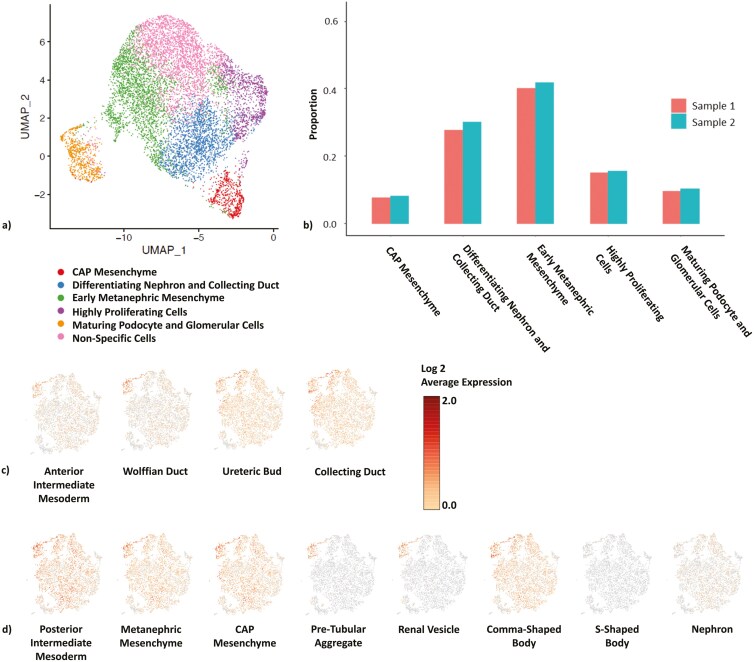
Single cell RNA sequencing analysis showed strong evidence for renal progenitor cell populations within starting organoids. (a) U-map clustering of cells shows statistically different populations of cells that can be loosely defined as CAP mesenchyme, differentiating nephron and collecting duct cells, early metanephric mesenchyme, highly proliferating cells, maturing podocyte and glomerular cells, and nonspecific cells based on cross-referencing genes of most statistical significance in each cluster (Table S1). (B) Evaluating the proportion of cells of each identified population in 2 different batches of organoids showed little variability between the cell proportionalities of the 2 batches of organoids. (c and D) TSNE plots showing Log 2 average expression of linage specific markers (Table 1) identified strong populations of cells with: (C) collecting duct lineage specific markers (developmental path progresses from anterior intermediate mesoderm, Wolffian duct, ureteric bud, to collecting duct), and (D) nephron linage specific markers (developmental path progresses from posterior intermediate mesoderm, metanephric mesenchyme, CAP mesenchyme, pre-tubular aggregate, renal vesicle, comma-shaped body, S-shaped body, to nephron).

Cell cluster stratification shows that at encapsulation, organoids have distinct clusters of cells that may be loosely defined as early metanephric mesenchyme, CAP mesenchyme, tubule and collecting duct, podocyte, and highly proliferating cells based on a literature review of statistically significant genes in each cluster (Figure 3a, Figure S9, Table S1). A proportionality analysis showed comparable proportions of cell clusters with little variability between our 2 different batches of organoids (Figure 3b). T-SNE plots identifying cells with well-known markers of real cells at each stage of development (Table 1) show that our starting organoids hold strong populations of collecting duct (Figure 3c) and nephron (tubule and glomerular) (Figure 3d) cell types.

**Table 1.** Markers for different stages of renal differentiation.

Collecting duct lineage							
Anterior intermediate mesoderm	Wolffian duct	Ureteric bud	Collecting duct				
HOXB4 HOXB7 LHX1 OSR1	HOXB7 LHX1 PAX2/8	HOXB7 EMX2 GATA3 LHX1 PAX2/8 RET GFRa1 WNT9B WNT11 FGF2/9 BMP7	HOXB7 GATA3 NOTCH2 Jag1 FOXi1 AQP2 AQP6 CALB1 SLC2614				
Nephron lineage							
Posterior intermediate mesoderm	Metanephric mesenchyme	Cap mesenchyme	Pre-tubular aggregate	Renal vesicle	Comma shaped body	S shaped body	Nephron
HOX10/11 OSR1 EYA1	HOX10/11 LHX1 PAX2/8 EYA1 OSR1	HOX11 OSR1 EYA1 PAX2 SIX1/2 GDNF	WNT4 FGF8 PAX8 LHX1	WNT4 FGF8 PAX8 LHX1 BRN1	WNT4 LHX1 Notch1/2	LHX1 KDR	LRP2 SYNPO NPHS1 UMOD CDH1 AQP1 LTL

These data confirm that nephron progenitor committed organoids capable of tubule, collecting duct, and glomerular tissue generation were encapsulated in hydrogel for the subsequent experiments in this study.

### Defined collagen-genipin scaffolds support the development of renal structures

Renal progenitor organoids have been documented to express enhanced development when encapsulated in a scaffold material.^20,21^ Having translation in mind, we sought to use a fully characterized biocompatible scaffold material that can be easily functionalized. Importantly, this scaffold material had to be injectable to allow for minimally invasive application into the renal parenchyma. The scaffold material we utilized consisted of a 0.2% rat tail type 1 collagen cross-linked with 0.25mM Genipin—a cross-linker 3000 fold less cytotoxic than glutaraldehyde.^22^ As the successful growth of renal organoids in such a scaffold has not been previously demonstrated, we followed and characterized the growth of our organoids in this scaffold. Assessment of viability, cell differentiation, and structure development of hydrogel-encapsulated organoids was performed via weekly live-dead assays, H&E staining, immunohistochemical (IHC) analysis, and scanning (SEM) and transmission (TEM) electron microscope imaging over a 4-week culture period.

**Figure 4.**
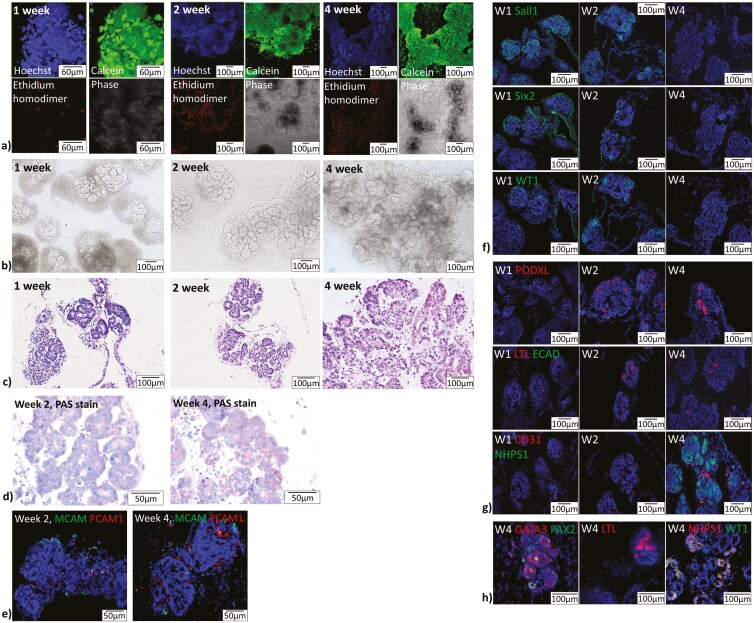
Analysis of 1-week, 2-week, and 4-week time points showed maturation of renal progenitor organoid micro-tissues. (A) Live/dead staining of 1-, 2-, and 4- week time points showed sustained high uptake of calcein AM, indicative of high viability, as well as increasing uptake of ethidium homodimer, indicative of dead cells, though comparatively less than calcein AM. Hoechst stain identifies staining regions as cells rather than debris. (B) Bright-field images of 1-, 2-, and 4-week timepoints showed progressive development of tubule-like structures, growth of organoids, and fusion of organoids into larger tissue structures. (C) H&E images of 1-, 2-, and 4-week timepoints support the findings of bright field imaging while giving further evidence of tubule-like structure formation. Luminal tubule-like structures showed increasing polarity with increasing duration of culture. (D) PAS staining comparing week 2 with week 4 samples showed increased basement membrane staining at the later timepoint, indicative of tissue maturation. (E) MCAM and PCAM1 staining shows higher MCAM at earlier time points, and higher PCAM1 staining at later time points, indicative of vascular maturation. (F) Early markers of renal development SALL1, Six2, and WT1 in green showed a decrease in expression over time from week 1 to week 4 postencapsulation. (G) Mature markers of renal development PODXL1, LTL, ECAD, CD31, and NHPS1 in red showed an increase in expression over time from week 1 to week 4 post-encapsulation. (H) Markers of different mature renal cell populations showed presence of collecting duct (GATA3, Pax2), proximal tubule (LTL), and glomerular (NHPS1, WT1) cell populations after 4 weeks in culture. Auto-fluorescence and secondary controls are shown in Figure S4.

Taken together, our findings show that long-term renal structure maturation is possible using the combination of our renal progenitor organoids and defined collagen-based scaffold. Live-dead staining showed high calcein AM uptake at all time points (Figure 4a). Ethidium homodimer uptake was increased over time as well, but the majority of cells appear to be viable (Figure 4a). Bright-field images showed the development of tubule-like structures with greater clarity starting at the 1-week time-point, with increasing organoid diameters and fusion with adjacent organoids beginning at 2 weeks (Figure 4b). By the 4-week time point, many organoids had fused to form larger tissue structures (Figure 4b). H&E images show clear luminal structures with increasing polarity in their structural organization starting at 2 weeks (Figure 4c). PAS staining comparing 2-week and 4-week time points shows a marked clarity of staining in the basement membrane at the later time point (Figure 4d). IHC images of MCAM and PCAM1 expression showed a higher expression of MCAM, identifying early vasculature, at 2 weeks and a higher expression of PCAM1, identifying more mature vasculature, at 4 weeks (Figure 4e). Furthermore, as early renal progenitor markers SALL1, Six2, and WT1 decreased in expression over time, mature renal markers PODXL, LTL, ECAD, CD31, and NHPS1 increased in expression (Figure 4f, g).

**Figure 5.**
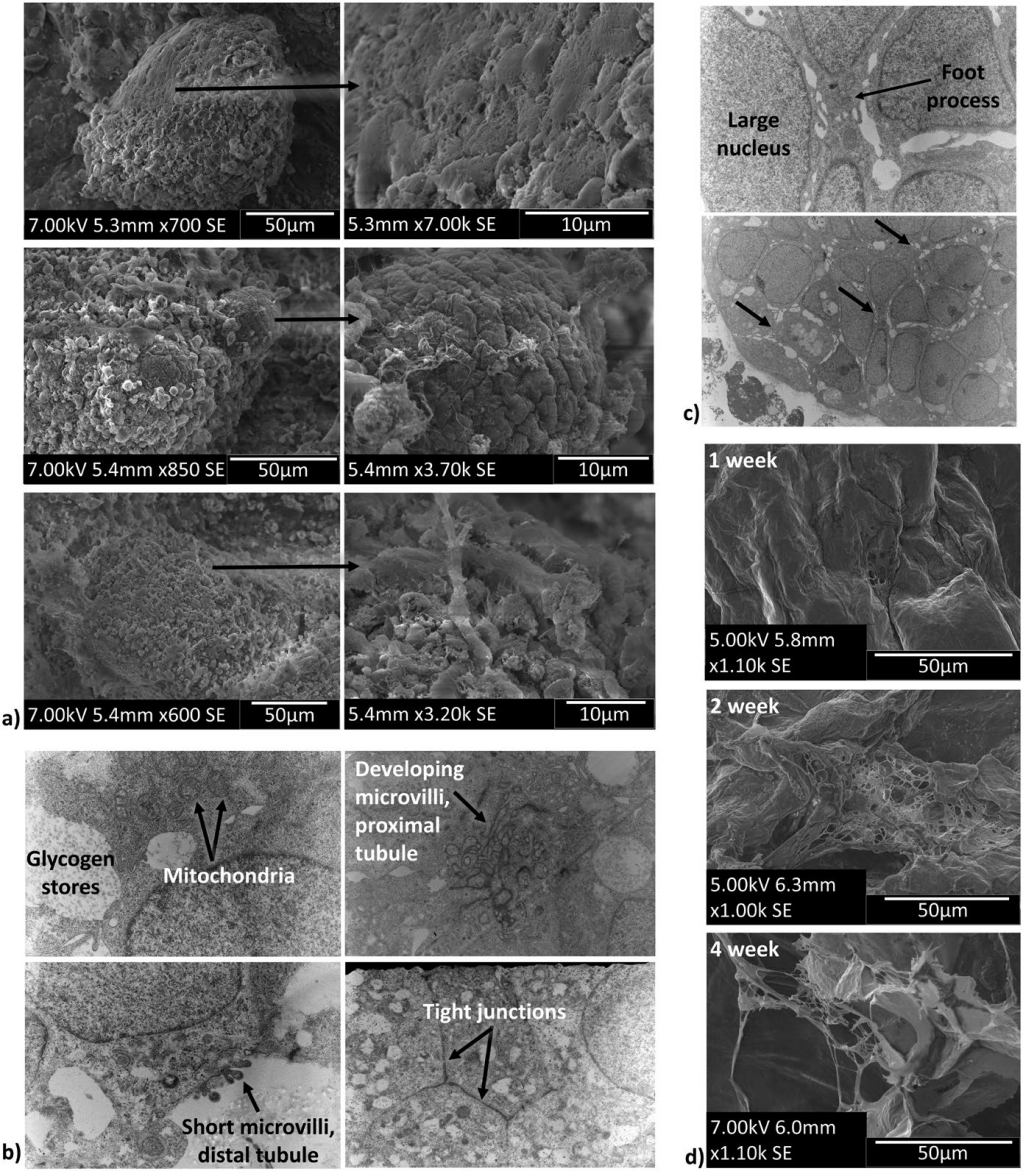
SEM and TEM imaging shows evidence of tubule-like and glomerulus-like structure formation. (A) SEM imaging of organoid structures at 4-week timepoints showed organoids with different morphological regions, including spherical cobble-stone like regions indicative of glomerular morphology. Branching vessel-like structures were also seen at this timepoint. (B) TEM evaluation of 4-week samples showed morphological features of tubule cells, including a high amount of mitochondria, large glycogen stores, tight junctions, developing long microvilli similar to proximal tubule cells, and short microvilli similar to distal tubule cells. (C) TEM evaluation also showed regions within organoids with podocyte-like cells with large nuclei and long processes that look similar to foot processes. (D) SEM evaluation of scaffold degradation showed marked degradation beginning at 2 weeks post-encapsulation and high porosity at 4 weeks post-encapsulation.

Week 4 timepoint sections were stained further for more specific markers of mature nephron cell populations. Images show the presence of collecting duct cells (GATA3+, PAX2+), proximal tubule cells (LTL+), and glomerular cells (NHPS1+, WT1+) (Figure 4h). SEM images at 4 weeks showed morphologically different cell populations within the same organoid, with some glomerular-like regions with a typical cobblestone appearance. Some organoids also showed branching vessel-like structures (Figure 5a). TEM images of 4-week scaffolds showed further evidence of organoid maturation into tubule-like structures with high numbers of mitochondria, glycogen stores, tight junctions, and developing microvilli (Figure 5b); and showed evidence of glomerulus formation with podocyte-like cells with large nuclei and appendages akin to foot-processes (Figure 5c).

Looking at the scaffold’s structure, SEM images of hydrogel constructs showed that organoid-laden scaffolds started degrading at approximately 2 weeks, forming roughly 15 µm in diameter pores. By 4 weeks, the scaffolds had degraded substantially, forming pores approximately 50 µm in diameter as the hydrogel was overtaken by tissue developed from organoid fusion (Figure 5d).

These data demonstrated that renal progenitor organoid viability, development, and maturation were possible in a controlled environment lacking the supportive factors within Matrigel.

### Minimally invasive implantation supported the integration and development of renal structures in vivo

**Figure 6.**
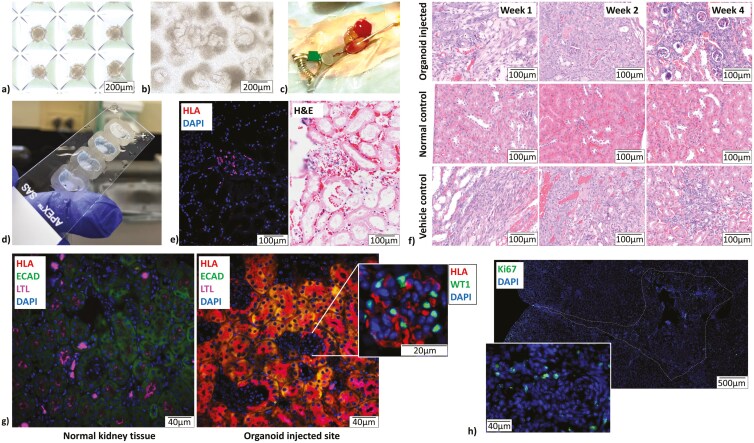
Renal progenitor organoids were successfully delivered, maintained, and integrated into sites of injection into tissue. (A) Organoids used for in vivo application are of a similar size and shape to those used for in vitro studies. They were also evenly spaced in micro-wells and of even size prior to collection for hydrogel encapsulation. (B) In vitro culture of organoids used for the surgical procedure, undergoing the same stresses, showed organoids that were able to mature as they had in previous in vitro experiments. (C) Injection of hydrogel into renal parenchyma can be seen clearly through tissue blanching. (D) Sections of day-2 post-injection kidneys clearly showed the retention of the scaffold at the injected site. (E) HLA staining and DAPI staining, along with H&E staining of day-2 post-injection kidney sections showed the presence of organoids retained at the injection site. (F) Images of H&E staining of 1-, 2-, and 4-week post-injection kidneys showed increased basophilic regions indicative of regeneration in both organoid-laden and vehicle control groups compared to time-matched normal kidneys. Organoid-laden injection groups showed an increased number of glomeruli in basophilic regions compared to the vehicle only control. (G) Organoid injected regions showed strong HLA staining in regions that stain positively for tubule (LTL and ECAD) and glomerular markers (WT1). (H) Ki67 staining at the 4-week timepoint showed the presence of a few proliferating cells remaining in the injection site.

We injected hydrogel-encapsulated organoids (5 days post-aggregation) into the renal cortex of athymic mice (Figure 6a). An estimated 2000 organoids were delivered per injection through a 23-gauge needle. Injection of hydrogels was successful, with minimal leakage after needle removal. Hydrogel placement was seen via regional blanching of the tissue during injection (Figure 6c). A gross examination of the kidney sections at 2 days post-injection showed scaffold regions in the renal cortex (Figure 6d). H&E and IHC staining for Human Leukocyte Antigen (HLA) to identify human-derived organoid cells in the setting of the mouse kidney in 2-day post-surgical kidney samples confirmed the retention of organoids at the injection site (Figure 6e). H&E staining across all timepoints showed that injected regions, both organoid-laden and organoid-free, produced basophilic regions indicative of regeneration and increased proliferation of cells compared to normal untreated tissue. Organoid-laden groups showed a larger number of developing glomeruli at the 4-week time point compared to vehicle-only controls (Figure 6f). IHC analysis of injection sites at 4-week time points showed regions of tissue that stain strongly for HLA markers (Figure 6g). These tissue regions also stained positively for tubule markers ECAD and LTL and glomerular marker WT1 in morphologically appropriate areas (Figure 6g). Ki67 staining at the 4-week timepoint showed that a low level of proliferation is ongoing at this later timepoint (Figure 6h).

Another group of samples from the same batch of organoids used in our in vivo experiments, undergoing the same procedures from organoid collection to passage through a 23-gauge needle were encapsulated and temperature cross-linked in 48-well plates on the day of surgery for in vitro culture. These samples were prepared to assess the possible effects of organoid processing for surgery. These samples showed structural maturation comparable to our other in vitro studies (Figure 6b).

## Discussion

### Overarching clinical significance

Our objective was to *demonstrate proof-of-concept that renal organoid technology can be used in a minimally invasive approach* to generate new renal tissue in the parenchyma—a clinically relevant site. We developed a straightforward, reproducible method to produce large quantities of renal progenitor organoids and tested a clinically feasible collagen-based delivery system for organoid maturation. Our results show that these organoids mature into structurally comprehensive renal tissue with tubule and glomerular microarchitecture. In vivo implantation demonstrated that organoid-derived structures expand into larger nephron-like tissues in the parenchyma. This system supports the progression of renal organoid technology toward clinical applications, addressing the limitations of current therapies.

Currently, dialysis is the only preventative therapy, and renal transplantation is the only definitive treatment for renal failure. However, both are limited by declining function and donor organ shortages. Renal transplantations, though lifesaving, are often not done until patients reach the point of ESRD, defined as 70% loss of kidney function. Additionally, it is expected that by 2030, the majority of renal failure cases will be in countries that lack the infrastructure to support current care options.^1^

As such, we focused on showing proof-of concept for a minimally invasive approach that does not require extensive infrastructure. In doing so, our approach not only supports the development of therapies that may address ESRD but also sets a precedent for developing *preventative treatments* for renal failure. Our minimally invasive approach could offer earlier intervention at stage 2 chronic kidney disease (60%-89% function remaining) by delivering viable kidney tissue to boost function and support tissue recovery. Furthermore, providing several seeds of regenerative tissue niches into a fibrotic tissue area may provide the cues necessary for native tissue to recover from damage, as recently demonstrated in other organ systems.^23^

Previous studies have demonstrated some success in using organoid technology to regenerate renal tissue in the parenchyma. However, the use of a 5/6 nephrectomy model for tissue implantation and limited cell sources presents challenges for clinical translation. For example, Harari-Stenberg et al showed the beneficial effects of boosting renal function and halting kidney disease progression in delivering nephron progenitor cells from human fetal kidneys in one study, and tubule cell aggregates in another.^18,24^ Similar to our findings, these studies highlight the importance of CAP mesenchyme progenitor cells and 3D aggregates in generating renal-like tissue, mostly focusing on the engraftment of tubule-like structures.^18,24^ As in these studies, our implanted cells also showed varied engraftment patterns with some evidence of single-cell integration within tubules, with the majority of our cells appearing to benefit from cell–cell interaction when engrafting, showing a pattern of grouped HLA + structure formation.^18,24^ Our study builds on these findings by using a more comprehensive source of renal progenitor cells that produces both tubule- and glomerulus-like tissue structure engraftment integrated into the native renal tissue. Unlike previous methods, our study employs a minimally invasive implantation method that preserves the renal capsule and demonstrates the successful engraftment of bioengineered real tissue into the high-pressure environment within an undisturbed capsule. This proof-of-concept study bridges the gap toward clinically viable organoid delivery therapies. Additionally, though iPSC technology needs better control and production methods, this starting cell source offers the potential to avoid lifelong immune suppression for patients.

Beyond the use of our approach in developing a direct treatment for patients, the easily scalable organoids we developed with more comprehensive nephron structures and a more defined supportive scaffold aid the future *development of more biomimetic*  *in vitro*  *drug testing* and developmental studies for kidney tissue.

## Organoid aggregation and delivery strategy

### In vitro studies

To advance renal organoid technology toward clinical translation, our in vitro studies focused on improving control over renal organoid development and maturation. We used an Aggrewell system to generate high yields of evenly sized small organoids with minimal loss (Figure 2). Previously developed methods, including transwell, 96-well, and bioreactor culture systems, generated renal organoids but were challenged by size control and lower yields.^6,8-10,19^ While smaller organoid batches may be sufficient for specific in vitro and small animal studies, larger tissue mass will be needed for translational applications. Furthermore, it was recently found that the most significant factor in the variability between generated organoids is the timing of the batch of differentiation.^25^ By easily producing more organoids from one batch of differentiation, we can achieve a more consistent product.

Additionally, our strategy of using smaller starting organoids with a diameter of roughly 194µm focuses on improving cell survival during in vitro and in vivo applications. Smaller organoid sizes support nutrient and metabolite diffusion, allowing for longer culture and maturation. We generated organoids rather than using dispersed cells for our studies also to allow better survival as providing pre-formed organoid structures with supporting microarchitecture and cell-to-cell crosstalk will allow for a more stable environment in the high-stress conditions of the renal parenchyma.

Another method employed to control renal organoid development involved using a fully characterized scaffold of 0.2% collagen cross-linked with 0.25 mM of Genipin. To enhance clinical translation, we selected a well-characterized biocompatible scaffold with predictable degradation, functionalization capacity, and injectability. Previous studies utilized Matrigel scaffolds to support renal progenitor cells and organoid development for 3D culture and in vivo delivery.^12,21^ However, as a protein mixture secreted by mouse sarcoma cells, Matrigel is ill-defined, batch-variable, and unsuitable for clinical studies.

Type 1 collagen is a well-defined, controlled scaffold material that is biocompatible and predictably degradable, widely used in tissue engineering.^26^ A collagen-based scaffold was favorable because we sought a material that remains fluid before injection, but cross-link quickly upon injection. Though collagen can temperature cross-link, adding a cross-linking compound was necessary to prevent short-term leaking. Hydrogels made with 0.2% type 1 collagen cross-linked with 0.25 mM of Genipin have been studied for other stem cell types and was also applied for cardiac in vivo studies, but not for renal applications.^22,27,28^ Furthermore, the temperature-dependent cross-linking properties of collagen and the time-dependent cross-liking properties of Genipin offer 2-step control of gelation, useful in unpredictable surgical settings.

Our results, like others, show that collagen injection alone can induce some regeneration in the kidney (Figure 6).^29^ Although this effect is small, functionalizing an injectable collagen scaffold to prolong or enhance these effects could open new avenues for effective in situ regenerative strategies in the kidney parenchyma. Genipin, a cross-linker that binds amine groups, offers a simple way to functionalize scaffolds with growth factors abundant in amine groups. Scaffold functionalization my provide more control and optimization of acellular scaffolds in future studies as specific bioactive compounds involved in kidney regeneration are identified.^30^

### In vitro findings

Although Aggrewell systems have been used for other organoid types, their use in renal organoid development is novel. Thus, we needed to demonstrate that renal cell types and structures could mature adequately using this system. Post-aggregation evaluation via bright-field, H&E, scRNAseq, and IHC analysis showed nephron-like structures at the implantation timepoint, including early tubule-like structures (Figure 2). ScRNAseq analysis indicates that our starting organoids are largely committed to renal lineage, with strong populations of CAP mesenchyme cells and ureteric bud cells that develop into nephron and collecting duct structures respectively upon maturation. Data also show a population of glomerular cells at the time of implantation (Figure 3), corroborated by IHC analysis with positive staining for early renal markers. The presence of podocyte and vascular cell populations is important here because these cells are known to release pro-angiogenic factors to vascularize tissues in renal development.^12,31,32^ Our organoids of a self-sustaining diameter may provide better conditions for angiogenic ingrowth and support the long-term survival of engineered constructs in the presence of these factors.

Since our scaffold material is of novel use for long-term maturation of renal progenitor cells or organoids, evaluating organoid viability and renal maturation within this non-enriched scaffold was necessary. Our results demonstrate the viability and proper development of nephron-like structures within our collagen-genipin hydrogel scaffold over time (Figures 4 and 5).

Week-4 mature organoids stain positively for tubular, glomerular, collecting duct, and vascular markers upon IHC analysis, with appropriate morphology evident in SEM and TEM imaging. The formation of *both* glomerulus- and tubule-like structures is crucial for addressing the problem of renal failure. In a functioning nephron, glomerular and tubule cells work together to balance fluid pressures and allow filtration. Addressing the burden of disease requires both components, especially the glomerular portion, as diabetes with glomerulosclerotic pathology causes 45% of renal failure cases. Replacing one nephron component without the other may cause improper function and rapid decline of implanted tissue.

### In vivo findings

Knowing organoids can form maturing nephron-like structures in vitro, we delivered approximately 2000 organoids in hydrogel per injection into the anatomically relevant site of the cortical renal parenchyma. Previous studies have delivered renal progenitor cells or organoids into subcutaneous or sub-capsular regions, and most recently into lymph nodes, but never successfully into the renal parenchyma within an intact capsule.^12-15,18,33-35^ We demonstrate successful organoid retention at injection sites and, most importantly, the formation and integration of tubule- and glomerulus-like structures derived from HLA positive organoid cells into the renal cortex (Figure 6). Our findings provide strong proof-of-concept for the suitability of this approach for renal regeneration—a novel successful strategy without significantly compromising the kidney capsule.^18,36^

## Remaining challenges and future directions

Though promising, our studies still face some challenges. The direct function of the renal-like structures generated from organoids is challenging to assess because there is currently no system to successfully vascularize these organoids in vitro to allow anatomically correct filtration functionality. Though vasculogenesis occurs in our organoids, it is nowhere near the level of vascularization seen in normal tissue. In development, angiogenesis accounts for most of the vascularization of nephron structures, and blood flow through these early structures allows for their proper development and maturation.^37,38^ Future studies that integrate an in vitro system of vascularization with our organoid-laden hydrogel may allow for a method of testing direct filtration function.

Furthermore, though more mature nephron-like tissue organization is seen in our in vivo results, it is difficult to assess the functional improvement that they impart accurately. In these studies, we are injecting organoids into normal kidneys to show proof-of-principle, but there is a necessity to test this therapy on a damaged kidney model. However, there is no easily accessible in vivo animal model for chronic kidney disease.^18,39-51^ Kidneys are also very good at augmenting their function to compensate for any damage or loss of function so essentially, one kidney needs to be removed, while the one remaining is damaged to create a model that can assess functional improvement. Unfortunately, this significantly impacts the lifespan of the test animal. Further studies in developing an adequate animal model for chronic kidney disease that preserves the renal capsule may allow for better testing of the functional improvement that our injection provides.

## Conclusions

In the present study, we showed the augmentation of renal progenitor organoid production using a novel method of aggregation, and the successful culture of renal-like structures within a clinically feasible collagen-based hydrogel. We developed a strategy for renal progenitor organoid delivery that sets a foundation for minimally invasive therapies in the future. Additionally, we showed that renal progenitor organoids, once in the renal parenchyma, are capable of maturing into renal-like structures that appear well-integrated into the surrounding native tissue. Our methods also provide the foundation for better in vitro applications of renal progenitor organoids in future studies. Further work is necessary to establish a safe and reliable strategy, but this work provides a strong foundation to address the pressing ethical imperative for new therapies for renal failure.

## Supplementary material

Supplementary material is available at *Stem Cells Translational Medicine* online.

szae078_suppl_Supplementary_Figures_1-9_Tables_1

## Data Availability

Data supporting the findings of this study are available upon request from the corresponding author.

## References

[1] United States Renal Data System. 2017 USRDS annual data report: Epidemiology of kidney disease in the United States. National Institutes of Health, National Institute of Diabetes and Digestive Kidney Diseases.

[2] Luyckx VA, Tonelli M, Stanifer JW. The global burden of kidney disease and the sustainable development goals. Bull World Health Organ. 2018;96:414-422D. 10.2471/BLT.17.206441 29904224 PMC5996218

[3] Harambat J, Ekulu PM. Inequalities in access to pediatric ESRD care: a global health challenge. Pediatr Nephrol. 2016;31:353-358. 10.1007/s00467-015-3263-7 26628281

[4] Kumar V, Jha V. End-stage renal disease care in South Asia: demographics, economics, and opportunities. Clin Nephrol. 2016;86 (2016):23-26. 10.5414/CNP86S103 27469146

[5] Xie Y, Bowe B, Mokdad AH, et al. Analysis of the Global Burden of Disease study highlights the global, regional, and national trends of chronic kidney disease epidemiology from 1990 to 2016. Kidney Int. 2018;94:567-581. 10.1016/j.kint.2018.04.011 30078514

[6] Takasato M, Er PX, Chiu HS, Little MH. Generation of kidney organoids from human pluripotent stem cells. Nat Protoc. 2016;11:1681-1692. 10.1038/nprot.2016.098 27560173 PMC5113819

[7] Morizane R, Miyoshi T, Bonventre JV. Concise review: kidney generation with human pluripotent stem cells. Stem Cells. 2017;35:2209-2217. 10.1002/stem.2699 28869686 PMC5805143

[8] Taguchi A, Nishinakamura R. Higher-order kidney organogenesis from pluripotent stem cells. Cell Stem Cell. 2017;21:730-746.e6. 10.1016/j.stem.2017.10.011 29129523

[9] Freedman BS, Brooks CR, Lam AQ, et al. Modelling kidney disease with CRISPR-mutant kidney organoids derived from human pluripotent epiblast spheroids. Nat Commun. 2015;6:8715. 10.1038/ncomms9715 26493500 PMC4620584

[10] Przepiorski A, Sander V, Tran T, et al. A simple bioreactor-based method to generate kidney organoids from pluripotent stem cells. Stem Cell Reports 2018;11:470-484. 10.1016/j.stemcr.2018.06.018 30033089 PMC6092837

[11] Przepiorski A, Crunk AE, Holm TM, et al. A simplified method for generating kidney organoids from human pluripotent stem cells. J Vis Exp. 2021;170:e62452. 10.3791/62452 PMC9006336 33938892

[12] Bantounas I, Ranjzad P, Tengku F, et al. Generation of functioning nephrons by implanting human pluripotent stem cell-derived kidney progenitors. Stem Cell Rep. 2018;10:766-779. 10.1016/j.stemcr.2018.01.008 PMC5918196 29429961

[13] Tran T, Lindström NO, Ransick A, et al. In vivo developmental trajectories of human podocyte inform in vitro differentiation of pluripotent stem cell-derived podocytes. Dev Cell. 2019;50:102-116.e6. 10.1016/j.devcel.2019.06.001 31265809 PMC6684316

[14] Sharmin S, Taguchi A, Kaku Y, et al. Human induced pluripotent stem cell-derived podocytes mature into vascularized glomeruli upon experimental transplantation. J Am Soc Nephrol. 2016;27:1778-1791. 10.1681/ASN.2015010096 26586691 PMC4884101

[15] van den Berg CW, Ritsma L, Avramut MC, et al. Renal subcapsular transplantation of PSC-derived kidney organoids induces neo-vasculogenesis and significant glomerular and tubular maturation in vivo. Stem Cell Rep. 2018;10:751-765. 10.1016/j.stemcr.2018.01.041 PMC5918682 29503086

[16] Tsujimoto H, Kasahara T, Sueta SI, et al. A modular differentiation system maps multiple human kidney lineages from pluripotent stem cells. Cell Rep. 2020;31:107476. 10.1016/j.celrep.2020.03.040 32268094

[17] Zhang D, Du X, Zhang X, et al. induction and. Exp Ther Med. 2020;20:1307-1314. 10.3892/etm.2020.8844 32742364 PMC7388233

[18] Harari-Steinberg O, Omer D, Gnatek Y, et al. Ex vivo expanded 3D human kidney spheres engraft long term and repair chronic renal injury in mice. Cell Rep. 2020;30:852-869.e4. 10.1016/j.celrep.2019.12.047 31968258

[19] Morizane R, Bonventre JV. Generation of nephron progenitor cells and kidney organoids from human pluripotent stem cells. Nat Protoc. 2017;12:195-207. 10.1038/nprot.2016.170 28005067 PMC5278902

[20] Garreta E, Prado P, Tarantino C, et al. Fine tuning the extracellular environment accelerates the derivation of kidney organoids from human pluripotent stem cells. Nat Mater. 2019;18:397-405. 10.1038/s41563-019-0287-6 30778227 PMC9845070

[21] Garreta E, Kamm RD, Chuva de Sousa Lopes SM, et al. Rethinking organoid technology through bioengineering. Nat Mater. 2021;20:145-155. 10.1038/s41563-020-00804-4 33199860

[22] Jeffords ME, Wu J, Shah M, Hong Y, Zhang G. Tailoring material properties of cardiac matrix hydrogels to induce endothelial differentiation of human mesenchymal stem cells. ACS Appl Mater Interfaces. 2015;7:11053-11061. 10.1021/acsami.5b03195 25946697 PMC4684185

[23] Wiśniewska J, Sadowska A, Wójtowicz A, Słyszewska M, Szóstek-Mioduchowska A. Perspective on stem cell therapy in organ fibrosis: Animal models and human studies. Life (Basel). 2021;11:1068. 10.3390/life11101068 34685439 PMC8538998

[24] Harari-Steinberg O, Metsuyanim S, Omer D, et al. Identification of human nephron progenitors capable of generation of kidney structures and functional repair of chronic renal disease. EMBO Mol Med. 2013;5:1556-1568. 10.1002/emmm.201201584 23996934 PMC3799579

[25] Phipson B, Er PX, Combes AN, et al. Evaluation of variability in human kidney organoids. Nat Methods. 2019;16:79-87. 10.1038/s41592-018-0253-2 30573816 PMC6634992

[26] Meyer M. Processing of collagen based biomaterials and the resulting materials properties. Biomed Eng Online. 2019;18:24. 10.1186/s12938-019-0647-0 30885217 PMC6423854

[27] Macaya DJ, Hayakawa K, Arai K, Spector M. Astrocyte infiltration into injectable collagen-based hydrogels containing FGF-2 to treat spinal cord injury. Biomaterials. 2013;34:3591-3602. 10.1016/j.biomaterials.2012.12.050 23414684

[28] Avila MY, Navia JL. Effect of genipin collagen crosslinking on porcine corneas. J Cataract Refract Surg. 2010;36:659-664. 10.1016/j.jcrs.2009.11.003 20362860

[29] Lee SJ, Wang HJ, Kim TH, et al. In situ tissue regeneration of renal tissue induced by collagen hydrogel injection. Stem Cells Transl. Med.. 2018;7:241-250. 10.1002/sctm.16-0361 29380564 PMC5788870

[30] Cho KS, Ko IK, Yoo JJ. Bioactive compounds for the treatment of renal disease. Yonsei Med J. 2018;59:1015-1025. 10.3349/ymj.2018.59.9.1015 30328315 PMC6192891

[31] Lim DS, Jackson JD, Atala A, Yoo JJ. Leading approaches to vascularize kidney constructs in tissue engineering. Engineering 2022;19:117-127. 10.1016/j.eng.2022.05.004

[32] Pleniceanu O, Harari-Steinberg O, Omer D, et al. Successful introduction of human renovascular units into the mammalian kidney. J Am Soc Nephrol. 2020;31:2757-2772. 10.1681/ASN.2019050508 32753400 PMC7790207

[33] Dekel B, Amariglio N, Kaminski N, et al. Engraftment and differentiation of human metanephroi into functional mature nephrons after transplantation into mice is accompanied by a profile of gene expression similar to normal human kidney development. J Am Soc Nephrol. 2002;13:977-990. 10.1681/ASN.V134977 11912257

[34] Xinaris C, Benedetti V, Rizzo P, et al. In vivo maturation of functional renal organoids formed from embryonic cell suspensions. J Am Soc Nephrol. 2012;23:1857-1868. 10.1681/ASN.2012050505 23085631 PMC3482737

[35] Francipane MG, Han B, Oxburgh L, et al. Kidney-in-a-lymph node: a novel organogenesis assay to model human renal development and test nephron progenitor cell fates. J Tissue Eng Regen Med. 2019;13:1724-1731. 10.1002/term.2924 31267702 PMC7099580

[36] Tan Z, Rak-Raszewska A, Skovorodkin I, Vainio SJ. Mouse embryonic stem cell-derived ureteric bud progenitors induce nephrogenesis. Cells. 2020;9:329. 10.3390/cells9020329 32023845 PMC7072223

[37] Little MH, Combes AN, Takasato M. Understanding kidney morphogenesis to guide renal tissue regeneration. Nat Rev Nephrol. 2016;12:624-635. 10.1038/nrneph.2016.126 27573726

[38] Takasato M, Little MH. The origin of the mammalian kidney: implications for recreating the kidney in vitro. Development. 2015;142:1937-1947. 10.1242/dev.104802 26015537

[39] Zhu XY, Urbieta-Caceres V, Krier JD, et al. Mesenchymal stem cells and endothelial progenitor cells decrease renal injury in experimental swine renal artery stenosis through different mechanisms. Stem Cells. 2013;31:117-125. 10.1002/stem.1263 23097349 PMC3528811

[40] Chade AR, Zhu X, Lavi R, et al. Endothelial progenitor cells restore renal function in chronic experimental renovascular disease. Circulation. 2009;119:547-557. 10.1161/CIRCULATIONAHA.108.788653 19153272 PMC2758066

[41] Eirin A, Zhu XY, Jonnada S, et al. Mesenchymal stem cell-derived extracellular vesicles improve the renal microvasculature in metabolic renovascular disease in swine. Cell Transplant. 2018;27:1080-1095. 10.1177/0963689718780942 29954220 PMC6158551

[42] Semedo P, Correa-Costa M, Antonio Cenedeze M, et al. Mesenchymal stem cells attenuate renal fibrosis through immune modulation and remodeling properties in a rat remnant kidney model. Stem Cells. 2009;27:3063-3073. 10.1002/stem.214 19750536

[43] Lee SR, Lee SH, Moon JY, et al. Repeated administration of bone marrow-derived mesenchymal stem cells improved the protective effects on a remnant kidney model. Ren Fail. 2010;32:840-848. 10.3109/0886022x.2010.494803 20662698

[44] Geng XD, Zheng W, Wu CM, et al. Embryonic stem cells-loaded gelatin microcryogels slow progression of chronic kidney disease. Chin Med J (Engl). 2016;129:392-398. 10.4103/0366-6999.176088 26879011 PMC4800838

[45] Tarng DC, Tseng WC, Lee PY, Chiou SH, Hsieh SL. Induced pluripotent stem cell-derived conditioned medium attenuates acute kidney injury by downregulating the oxidative stress-related pathway in ischemia-reperfusion rats. Cell Transplant. 2016;25:517-530. 10.3727/096368915X688542 26132529

[46] Sedrakyan S, Villani V, Da Sacco S, et al. Amniotic fluid stem cell-derived vesicles protect from VEGF-induced endothelial damage. Sci Rep. 2017;7:16875. 10.1038/s41598-017-17061-2 29203902 PMC5715019

[47] Thiel A, Yavanian G, Nastke MD, et al. Human embryonic stem cell-derived mesenchymal cells preserve kidney function and extend lifespan in NZB/W F1 mouse model of lupus nephritis. Sci Rep. 2015;5:17685. 10.1038/srep17685 26628350 PMC4667213

[48] Quimby JM, Borjesson DL. Mesenchymal stem cell therapy in cats: current knowledge and future potential. J Feline Med Surg. 2018;20:208-216. 10.1177/1098612X18758590 29478398 PMC10816289

[49] Feng J, Lu C, Dai Q, Sheng J, Xu M. SIRT3 facilitates amniotic fluid stem cells to repair diabetic nephropathy through protecting mitochondrial homeostasis by modulation of mitophagy. Cell Physiol Biochem. 2018;46:1508-1524. 10.1159/000489194 29689547

[50] Lee RH, Seo MJ, Reger RL, et al. Multipotent stromal cells from human marrow home to and promote repair of pancreatic islets and renal glomeruli in diabetic NOD/SCID mice. Proc Natl Acad Sci U S A. 2006;103:17438-17443. 10.1073/pnas.0608249103 17088535 PMC1634835

[51] Ezquer F, Giraud-Billoud M, Carpio D, et al. Proregenerative microenvironment triggered by donor mesenchymal stem cells preserves renal function and structure in mice with severe diabetes mellitus. Biomed Res Int. 2015;2015:164703. 10.1155/2015/164703 26167475 PMC4475763

