# Supplementary material for: Development and intra-renal delivery of renal progenitor organoids for effective integration in vivo
Source: Stem Cells Transl Med. 2024 Oct 28;14(1):szae078. doi: 10.1093/stcltm/szae078 (PMC11832275; doi:10.1093/stcltm/szae078)
Supplement: szae078_suppl_Supplementary_Figures_1-9_Tables_1 [file szae078_suppl_supplementary_figures_1-9_tables_1.pdf]

Supplemental information

Detailed methods and materials

1. Organoid preparation:

1.1 iPSC maintenance
Undifferentiated HiPSCs (ATCC ACS-1019 or Allen Institute WT11) were cultured on 6cm Cell Basement Matrix (ATCC) coated cell culture plates in discrete colonies until 80% confluence using Pluripotent Stem Cell Serum-Free Media (ATCC), supplemented with 10µM ROCK inhibitor Y27632 (Millipore Sigma) only on the first day of plating. The media was changed every day for 4-5 days. Cell colonies were evaluated using a phase-contrast microscope daily for the presence of differentiated cells and cleaned.

1.2 iPSC differentiation in 2D culture
The method of iPSC differentiation in 2D culture was adapted from an established method published by Takasato et al.[1]. HiPSCs expanded in 6cm plates were washed twice with Dulbecco's PBS and incubated with 3ml of TrypLE Select for 4 minutes at 37°C. Five ml of DMEM F:12 was added to the plates and the cell colonies were collected and centrifuged at 400G for 3 minutes. the supernatant fluid was aspirated and cells were re-suspended in fresh serum-free media with 10µM ROCK inhibitor Y27632 and plated at a density of 1.0 x 10^6 /cm^2 onto a 10cm cell basement matrix coated plate. After 1 day of incubation, serum-free media was removed and replaced with 8µM CHIR99021 (Tocris bioscience) in serum-free APEL medium (StemCell Technologies) for 4 days, followed by APEL medium containing 200ng/mL FGF9 (R&D systems) and 1µg/mL heparin (Sigma Aldrich) for 3 days to induce differentiation into intermediate mesoderm cells. The medium was changed every 2 days. Proper differentiation into intermediate mesoderm cells (early renal progenitors) was confirmed by staining plates with GATA3 (1:300) and HOXD11 (1:150) (Invitrogen).

1.3 Renal progenitor cell aggregation and 3D culture
Renal progenitor cells from 2D culture were detached using dispase (StemCell Technologies) and re-suspended in 8µM CHIR99021 in serum-free APEL media and incubated for 1 hour in a non-adherent plate. Cells were re-suspended in APEL medium containing 200ng/mL FGF9 and 1µg/mL heparin and distributed evenly into wells of an aggrewell plate (StemCell Technologies) at a density of 500 cells per micro-well and centrifuged. The FGF9-heparin medium was replaced every 2 days for 5 days to allow for cell aggregation. Organoids were collected from the Aggrewell plates using a 37µm reversible strainer, and collected organoids were re-suspended in APEL media for counting. The organoid yield was estimated by pipetting 50µl of the organoid suspension into a flat-bottomed 96-well plate, counting the number of organoids per well, and using the calculation below as suggested on the StemCell Technologies website:

$$Total\ number\ of\ organoids = \frac{organoids\ in\ 50\mu l}{50\mu l} \times volume\ of\ organoid\ suspension\ (\mu l)$$

Once counted, organoids were re-suspended in the hydrogel mixture as described below in section 2.2.

The average diameter of organoids at different time points was measured by taking bright field images of 4 quadrants in each of the six wells within the aggrewell plates, measuring each

organoid diameter using CellSense software, and averaging the values. The number of cells per organoid on the embedding day was estimated by dissociating and counting cells from a sample of organoids from the same batch. Accutase was applied to organoids and agitated every 30 minutes over 3 hours at 37°C. The cells were counted and divided by the number of organoids collected. This cell aggregation process and evaluation were repeated 15 times for this study.

2. Single Cell RNA sequencing

2.1 Organoid dissociation and preparation for sequencing

Organoids were prepared in two batches (cells thawed from different vials of iPSCs and differentiation started at different times) using the differentiation methods listed above. After 5 days of incubation, organoids were collected and washed with DPBS by centrifuging 300G for 4 minutes. After washing, organoids were enzymatically dissociated using a 1:1 mixture of DPBS and TrypLE Select at 37°C for 30 minutes. Digestion was stopped by adding complete media. Organoids were then pipetted to mechanically break apart clusters. The resulting cells were then centrifuged at 400G for 5 minutes and re-suspended in complete media.

Debris was removed using a 70 µm cell strainer (Corning). The cell strainer was flushed 3 times with 5mL media to ensure cells had passed through. The collected cells were counted.

To ensure high viability of cells for sequencing, a dead cell removal kit was used (Miltenyi Biotech). Our methods were adapted from the manufacturer's protocol by adding double filtration and additional washes using MACS buffer solution. Following cell collection, the MACS buffer was removed via centrifugation at 400G for 5 minutes. Cells were re-suspended in warm complete media and counted using a cell counter (Countess 3 Invitrogen) to confirm high viability (91% viability with 7.52 x 10^6 live cells/mL and 92% viability with 5.72 x 10^6 live cells/mL for the two samples used).

2.2 Sequencing and analysis

Cellular suspensions were loaded on a Chromium Single Cell Controller (10x Genomics, Pleasanton, CA, USA) to generate single-cell GEMs. Single-cell RNA-Seq libraries were prepared using Chromium™ Next GEM Single Cell 3' Kit v3.1 (10x Genomics, P/N 1000268). GEM-RT was performed in a Bio-Rad Thermal cycler with TempAssure PCR 8-tube strip (USA Scientific, P/N 1402-4700): 53 °C for 45 min, 85 °C for 5 min; held at 4 °C. After RT, GEMs were broken and the single-strand cDNA was cleaned up with DynaBeads MyOne Silane Beads (Thermo Fisher Scientific, P/N 37002D) and SPRIselect Reagent Kit (Beckman Coulter, P/N B23318). cDNA was amplified using the Bio-Rad Thermal cycler with TempAssure PCR 8-tube strip: 98 °C for 15 seconds, 63 °C for 20 seconds, and 72 °C for 1 minute; 72 °C for 1 minute; held at 4 °C. The amplified cDNA product was cleaned up with the SPRIselect Reagent Kit (0.6 × SPRI) and then the cDNA is fragmented. Indexed sequencing libraries were constructed using the reagents in Chromium™ Next GEM Single Cell 3' Kit v3.1 (10x Genomics, P/N 1000268) following these steps: (1) end repair and A-tailing; (2) adapter ligation; (3) post-ligation cleanup with SPRIselect; (4) sample index PCR and cleanup. The barcode sequencing libraries were quantified using the Qubit 3.0 (Thermo Fisher, USA) and Agilent TapeStation 4200 (Agilent Technologies). Sequencing libraries were loaded at 260 pM final concentration on an Illumina NovaSeq 6000 with SP-100 cycle kit (Illumina, P/N 20028401), paired-end sequencing using the

following read length: 28 bp Read1, 10 bp i7 Index, 10 bp i5 Index and 90 bp Read2. 10x Genomics analysis platform Cell Ranger was used to process the data.

UMAP and t-SNE renderings of the Cell Ranger analysis were used to identify different clusters of cells within our samples. Up-regulated genes per cluster were identified and a literature review was performed to cross-reference each upregulated gene to associate these cell clusters with likely cell types.

3. Vehicle of delivery preparation and evaluation

3.1 Collagen hydrogel preparation
Collagen hydrogel was prepared by combining ice cold 1x phosphate buffered saline, 1N NaOH, and soluble rat-tail type1 collagen in acetic acid to obtain a 2mg/ml collagen solution at pH 7.5. A stock solution of filtered Genipin (Wako Pure Chemical, Japan) diluted in 5x PBS to a concentration of 3mg/ml was made 1 hour before use, and was mixed into the collagen solution to achieve a Genipin concentration of 0.25mM. For *in vitro* studies, 200µl/well of hydrogel was mixed with and without organoids in a 48-well plate and incubated at 37°C to induce thermal gelation. For *in vivo* experiments, organoid-laden collagen gels were kept on ice until the surgical site was prepared. Genipin was added to the gel immediately before injection to achieve a concentration of 0.25mM.

3.2 Scanning electron microscopy (SEM) for evaluation of *in vitro* hydrogel degradation
Organoid-laden hydrogels and organoid-free hydrogels were cultured for 1-, 2-, and 4-week time points. These hydrogels were retrieved and washed prior to freezing at -80°C overnight and placed in a lyophilizer for 2 days. The dried hydrogels were critical point dried (Leica EM CPD300), sputter-coated (Leica EM ACE600), and imaged with a scanning electron microscope (Hitachi FlexSEM 1000) at an accelerating voltage of 5.00kV and 7.00 kV. Average pore sizes were measured for each sample.

3.3 Assessment of cell viability in collagen-genipin hydrogel
Cell-laden hydrogels were washed twice with DPBS. The DPBS was aspirated and replaced with 1ml of DMEM F:12 medium mixed with 0.5µl Calcein AM, 2µl Ethidium homodimer, and 1µg/ml Hoechst stain (Invitrogen) for live/dead staining. The entire gel was imaged using confocal microscopy (Olympus Flowview FV10i).

4. Evaluation of organoid development in vitro and integration in vivo

4.1 Sectional evaluation of organoids:
Preparation of pre-encapsulation organoids for staining: Organoids from day 5 post-aggregation were removed from aggrewell plates and centrifuged at 400G for 5 minutes. They were then re-suspended, fixed in 4% PFA in PBS for 20 minutes at -4°C, and washed with PBS 3 times. Several organoids were embedded in 0.2% agarose in discs and processed for paraffin embedding. This set of organoids was indicated as week 0 organoids in the following analyses.
Preparation of organoids matured in hydrogel for staining: Organoid-laden hydrogels from 1-week, 2-week, and 4-week culture time-points were fixed in 4% PFA in PBS for 30 minutes at -4°C and washed with PBS 3 times. The hydrogels were processed for paraffin embedding.
Staining: Paraffin blocks were sectioned at a 5µm thickness for staining. Antigen retrieval and deparaffinization was performed, followed by treatment with 0.2% Triton-X and protein

blocking for 30 minutes. Primary antibodies were incubated overnight at -4°C and secondary antibodies were incubated for 30 minutes at room temperature. The following antibodies and dilutions were used: Sall1 1:200 (ThermoFisher), Six2 1:100 (ThermoFisher), GATA3 1:300 (ThermoFisher), Pax2 1:2500 (ThermoFisher), LTL 1:300 (Vector Labs), ECAD 1:300 (SantaCruz), UMOD 1:1000 (Abcam), WT1 1:1500 (ThermoFisher), NHPS1 1:300 (Abcam), CD31/PCAM1 1:50 (Gilson), PODXL 1:300 (R&D Systems), CD146/MCAM 1:500 (ThermoFisher), HLA 1:150 (Abcam), LHX1 1:150 (Abcam), HOXD11 1:150 (ThermoFisher), Ki67 1:200 (Fisher Scientific). Images were taken using Olympus BX63 and Leica DM4000B fluorescence microscopes. All immunofluorescence analyses were repeated 8 times and representative images were presented. Hematoxylin and eosin (H&E) staining was also completed for each set of time points for morphological evaluation.

4.2 Confocal imaging of organoids in hydrogel:
Hydrogels were fixed with 10% neutral buffered formalin for 20 minutes at room temperature followed by a PBS wash. They were blocked with Dako protein blocking solution for 30 minutes at room temperature and incubated with primary antibodies overnight at -4°C. Secondary antibodies were incubated for 1 hour at room temperature. Images were taken using an Olympus Flowview FV10i confocal microscope. All immunofluorescence analyses were repeated 7 times and representative images were presented.

4.3 SEM and TEM imaging of organoid-laden hydrogels
SEM imaging: Organoid-laden hydrogels were cultured for 1-, 2-, and 4-week time points. These hydrogels were retrieved and washed prior to freezing at -80°C overnight and put in a lyophilizer for 2 days. The dried hydrogels were critical point dried, sputter-coated, and imaged with a scanning electron microscope with an accelerating voltage of 7.00 kV.
TEM imaging: Organoid-laden hydrogels were cultured for 1-, 2-, and 4-week time points. These hydrogels were retrieved and washed prior to fixing with 2.5% glutaraldehyde in 0.1M Millonig's phosphate buffer pH 7.3 for a minimum of one hour. Samples were washed three times in buffer and post-fixed with 1% osmium tetroxide in phosphate buffer for one hour. After washing in buffer, samples were dehydrated through a graded series of ethanol (10 minutes each) and samples were incubated in propylene oxide for two changes of 15 minutes each in preparation of resin infiltration. Finally, the samples were gradually infiltrated with 1:1, 1:2 and pure solutions of Spurr's resin after which they were allowed to cure in a 70ºC oven overnight. 90 nm sections were obtained with a Reichert-Jung Ultracut E ultramicrotome, stained with lead citrate and uranyl acetate, and viewed with a FEI Tecnai Spirit TEM operating at 80 kV. Images were obtained with an AMT 2Vu CCD camera.

4.4 *In vivo* collagen-genipin injection study
Two groups of hydrogel were prepared for implantation via injection: a control group organoid-free 0.2% collagen hydrogel cross-linked with Genipin, and an experimental group organoid-laden 0.2% collagen hydrogel cross-linked Genipin mixed in as described above. Organoids encapsulated in collagen-based hydrogel were prepared by re-suspending in 50µl of the hydrogel vehicle at a density of 2,000 organoids per injection and kept on ice until needed. The animals were divided into and sacrificed at 4 time points: 2-day, 1 week, 2 weeks, and 4 weeks (n=1 control and n=4 experimental groups for each time point. The left kidney was injected with organoid-laden hydrogel, and the right kidney was injected with hydrogel only control). Organoids encapsulated in the collagen-based hydrogel were injected into the kidneys of 6-8

week-old male athymic mice (Charles River). All animal procedures were performed in accordance with the NIH Guide for the Care and Use of Laboratory Animals using a protocol approved by the Institutional Animal Care and Use Committee at Wake Forest University Health Sciences (protocol number: A19-184). Animals were anesthetized with isoflurane and the left kidney was exposed via a ventral abdominal incision. A bulldog clamp was used to clamp the renal pedicle to prevent flushing of the hydrogel while it cross-linked post-injection. Organoid-laden hydrogels were injected at a volume of 50μl using a 23-gauge needle into the cortical region of the renal parenchymal, for the implant to be placed midline on the lateral margin of the kidney. Once the hydrogel was ejected from the syringe, the needle was kept in place for 1 minute to prevent leakage upon needle removal. Following needle removal, the bulldog clamp was removed and any active bleeding was controlled, followed by wound closure.
The kidneys were retrieved and fixed in 10% neutral buffered formalin, processed, and paraffin-embedded for sectioning at a 5μm thickness for staining. Staining and imaging was done as in section 3.2 above.

5. Statistical analyses

Descriptive statistical analysis was performed to describe the variability of organoid size, organoid number, and organoid production by an expression of mean ± standard error of the mean.

Analyses of single cell RNA sequencing data are discussed above in section 2.2 of the methods.

<u>Supplemental figures</u>

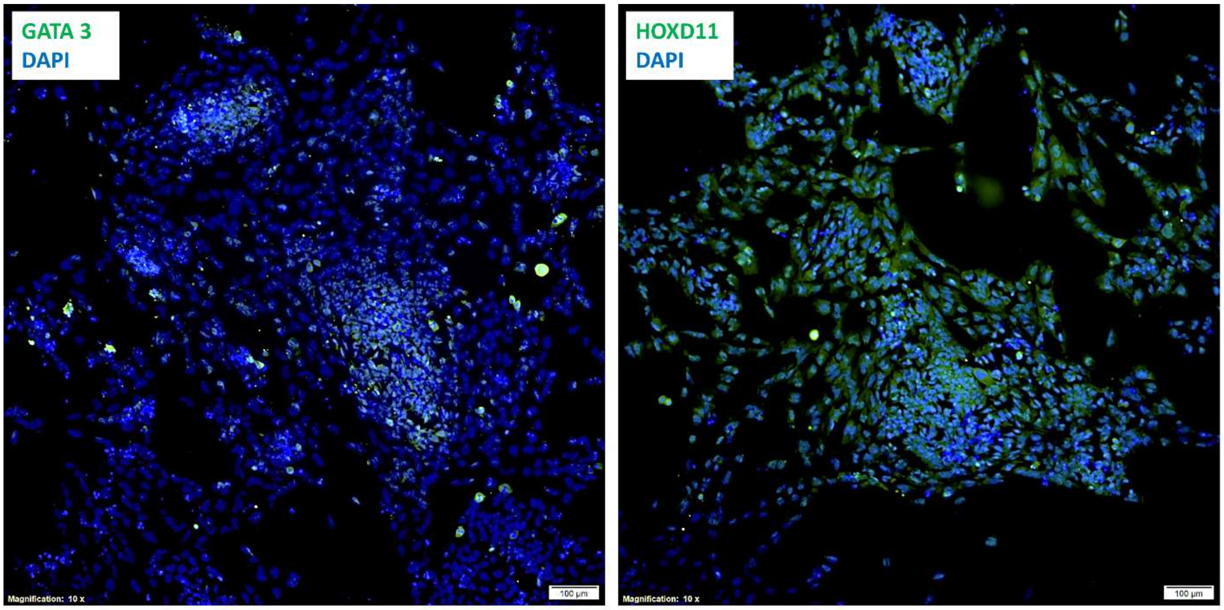

Supplemental figure 1: **Renal progenitor cell populations post-differentiation in 2D culture.** Cells treated with CHIR for 4 days and FGF9 for 3 days were fixed and stained to identify renal progenitor cell populations. GATA3 is indicative of ureteric epithelium cells that will later develop into collecting duct cells. HOXD11 is indicative of metanephric mesenchyme cells that will later develop into nephron cells.

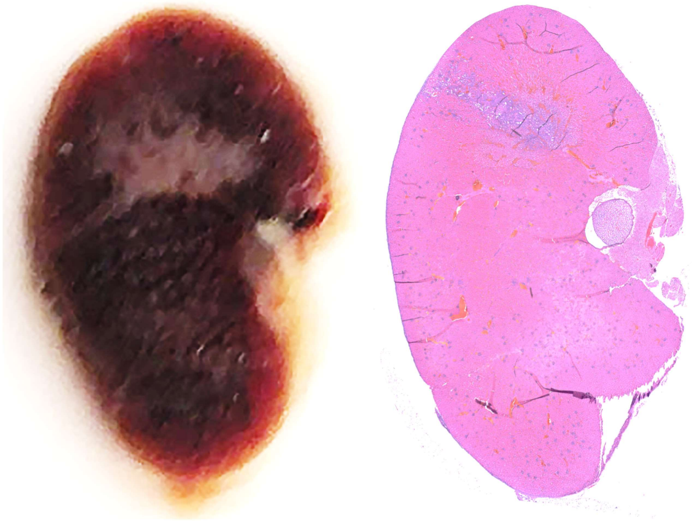

Supplemental figure 2: Results of hydrogel formulation testing. Gross paraffin section image (left) and H&E image (right) showing the localization of the injected scaffold material and resulting basophilic tissue 2 days post-surgery only at the upper pole where the Genipin cross-linked hydrogel was injected. No evidence of scaffold retention at the lower pole of the same kidney where collagen-only hydrogel was injected.

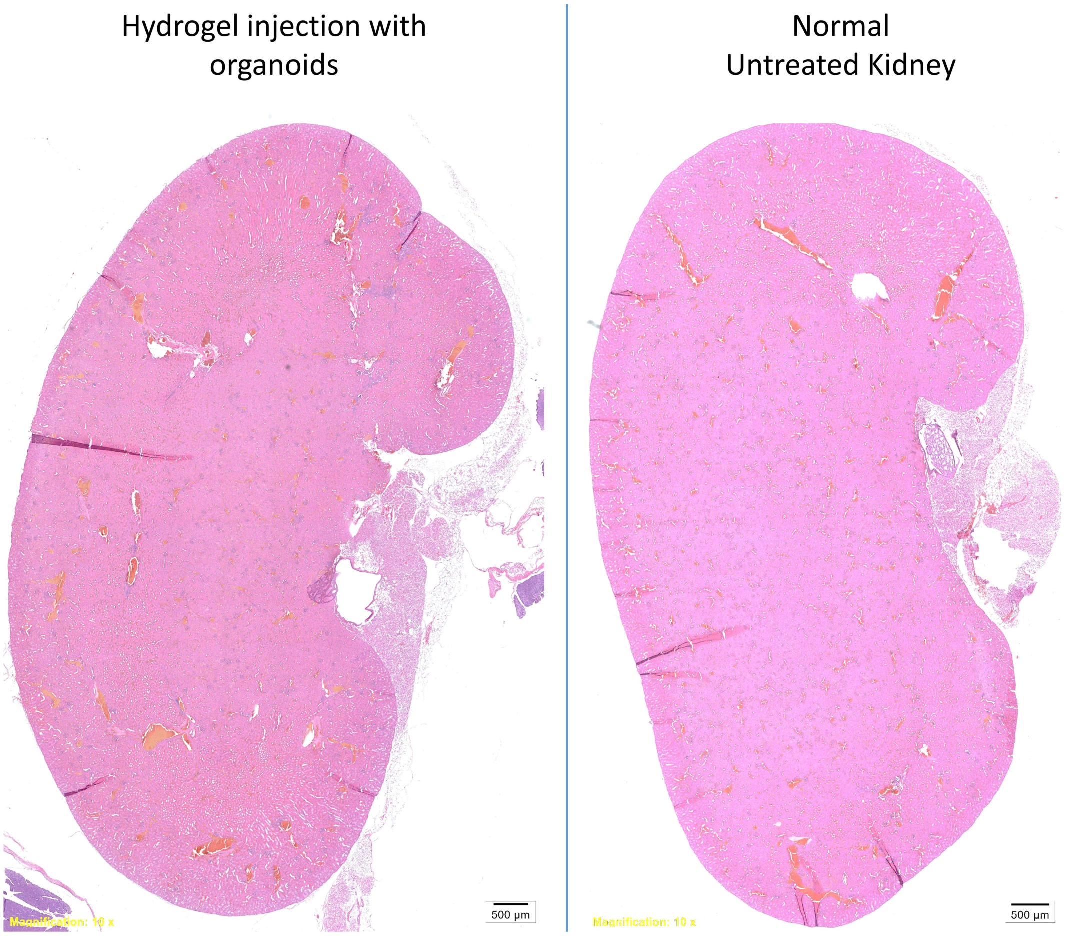

Supplemental figure 3: **Larger view H&E.** H&E images kidneys collected 4-weeks post-surgery.

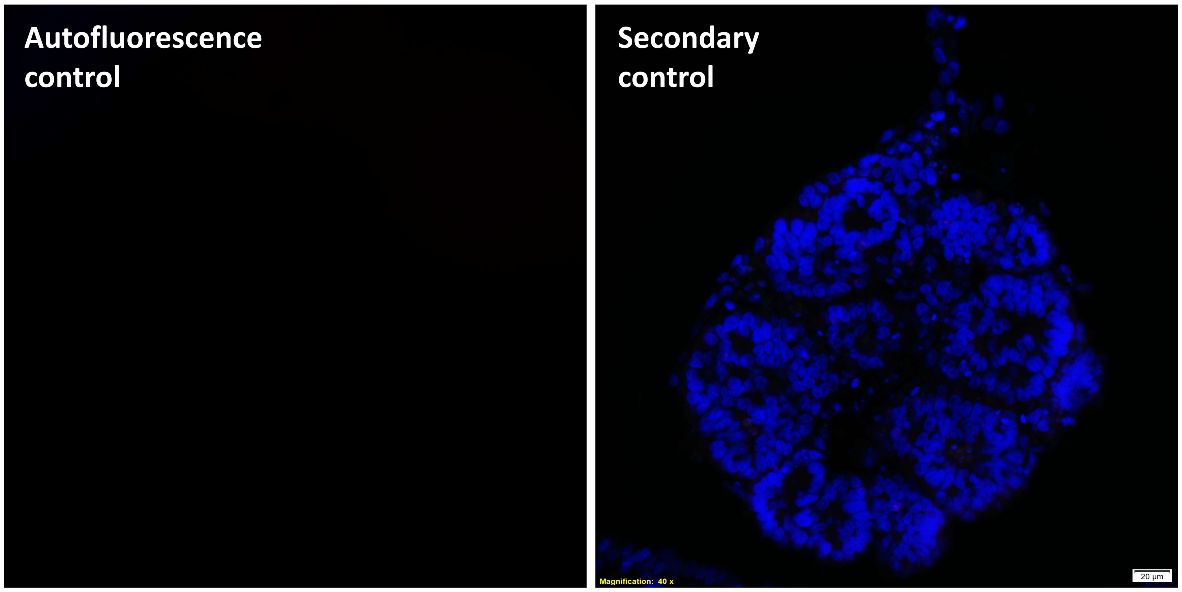

Supplemental figure 4: Auto-fluorescence and secondary controls for figure 2g and figure 3. The mounting media in the secondary control had DAPI in it to identify cellular regions.

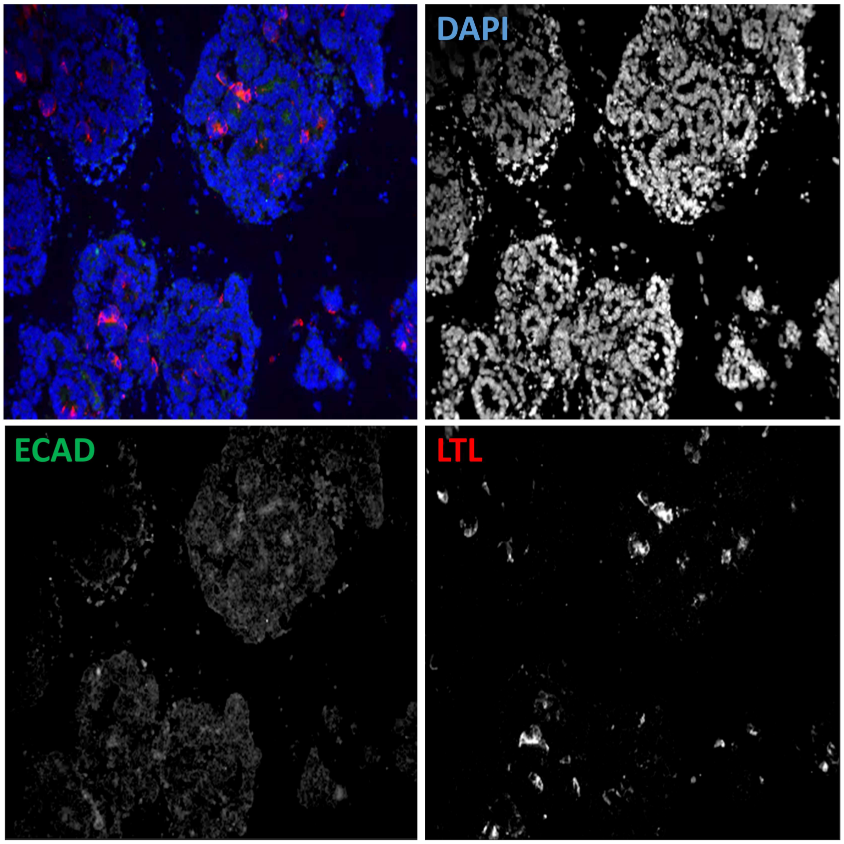

Supplemental figure 5: Images of channels separated for figure 4g, week 4 organoids.

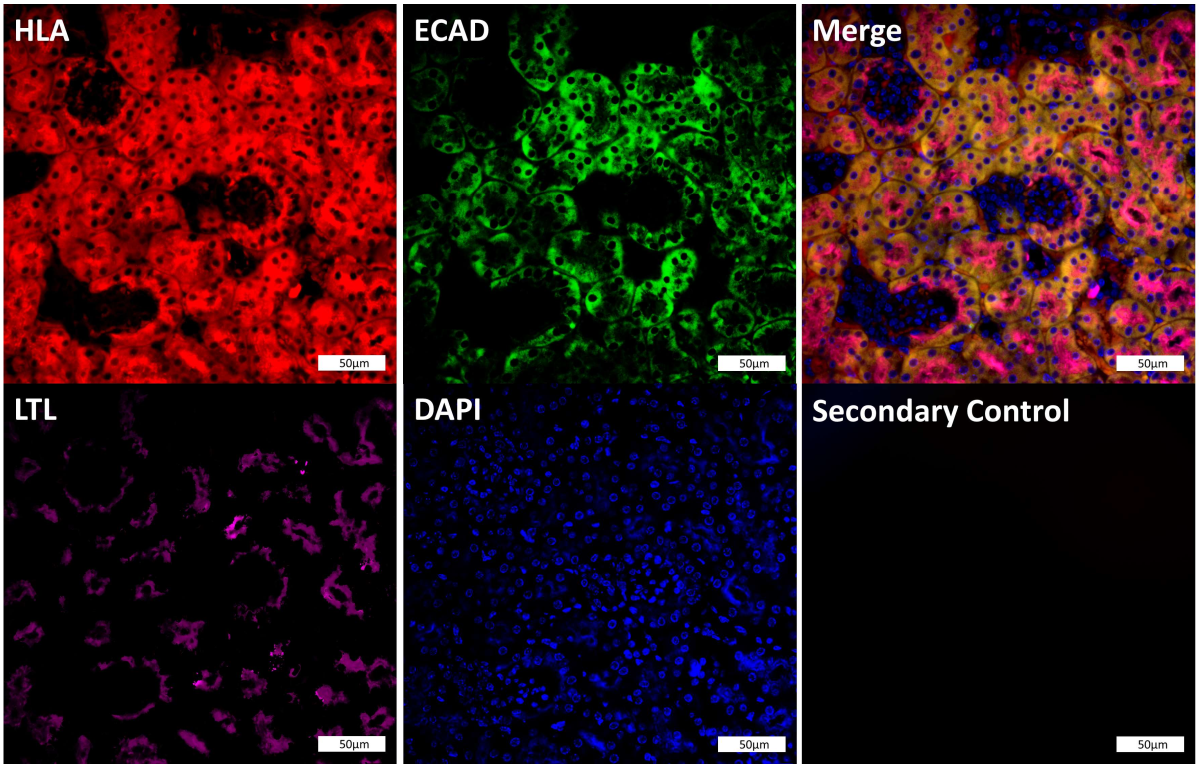

Supplemental figure 6: Images of channels separated for figure 6g kidney sections.

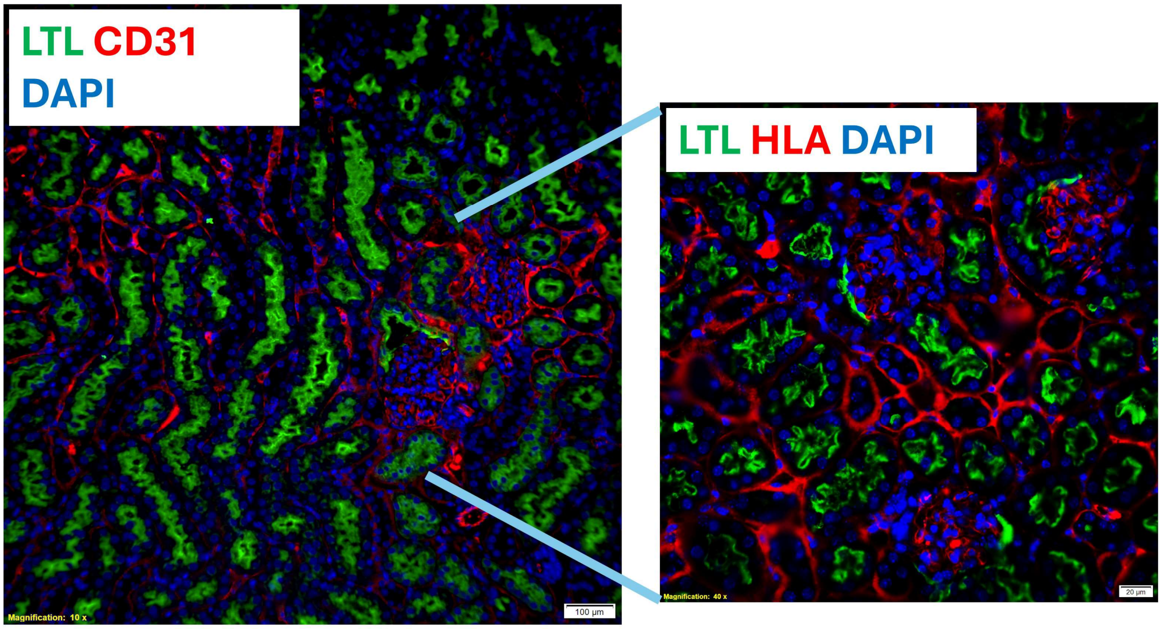

Supplemental figure 7: Images of CD31+ cells in HLA+ injected kidney regions 2 weeks post-surgery.

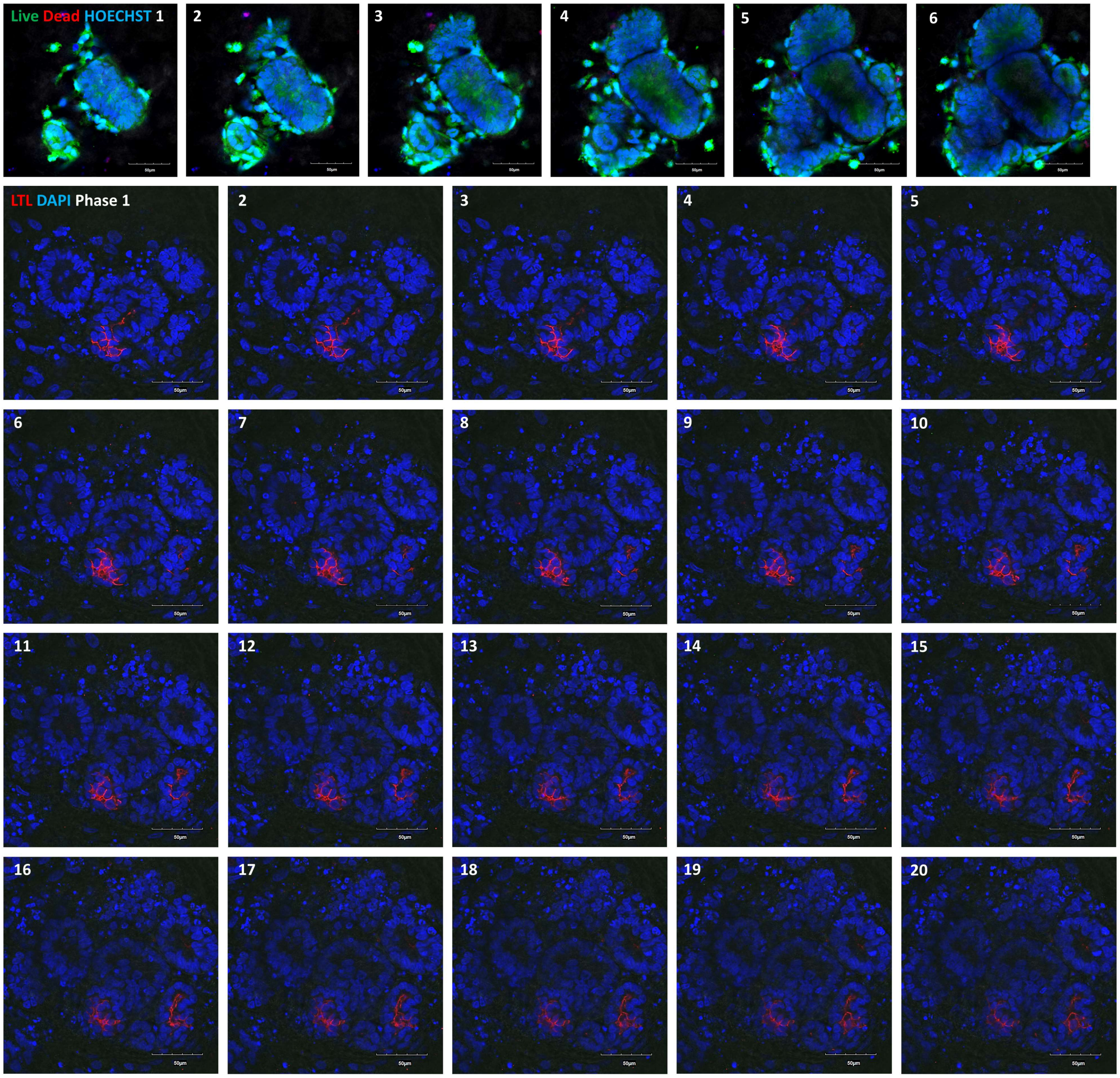

Supplemental figure 8: Confocal images of 4-week organoids showing tubule-like structures. Top row shows a Z-stack series of live-dead images. Bottom Z-stack images show LTL+ tubule-like structures.

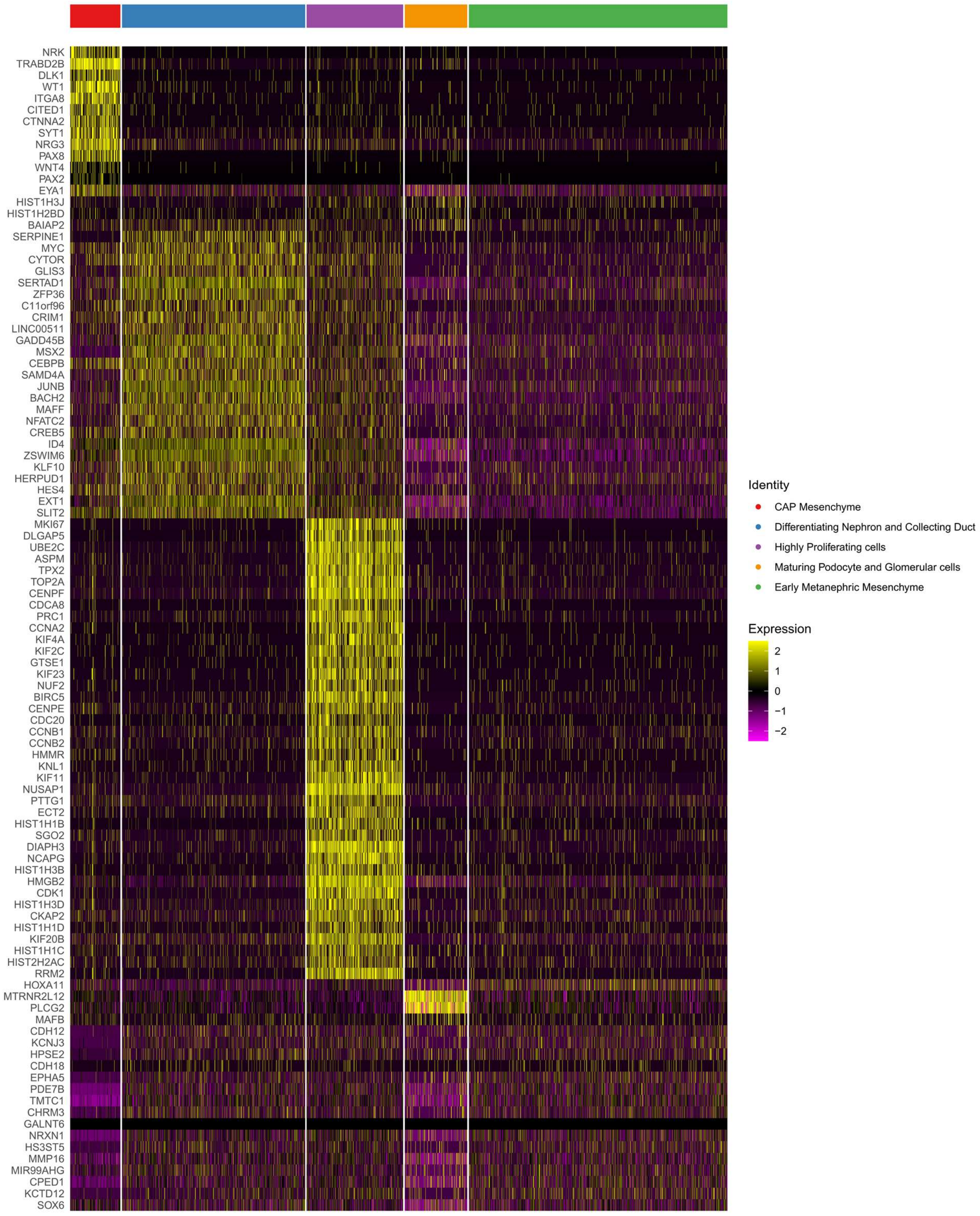

Supplemental figure 9: Heat map identifying cell clusters in organoids. Heat map of up-regulated genes that stratify cells within our organoid into distinct populations of cells. Genes of most significance were identified and cross-referenced to identify corresponding cell types in each cluster (**Table 1**).

**Table 1: Gene expression and cell cluster categorization of implanted organoids.**			
Gene	Function/relation to kidney component	P-value	Reference
Early Metanephric Mesenchyme			
HOX11A	Identifies early metanephric mesenchyme cell population	8.40e-2	[2-4]
Differentiating cells of nephron and collecting duct			
Serpine1	Identifies kidney tubule cell	1.43e-24	[5]
Myc	Modulator of renal organogenesis through regulation of progenitor cell population	1.79e-16	[6]
Cytor	Promotes EMT and cell proliferation	7.06e-13	
GLIS3	Identifies primary cilia tubules, an indicator of cell differentiation	4.39e-12	[7]
SERTAD1	Podocyte expression but also expressed in other organ systems as well	5.55e-12	[8]
ZFP36	Proliferation inhibitor	4.17e-11	[9]
C11orf96	Highly expressed in the kidney, mainly in glomerular epithelial cells, and has a role in renal tubule formation	8.08e-11	[10]
CRIM1	Regulates release of VEGFA by podocytes during renal organogenesis	1.22e-10	[11]
LINC00511	Involved in the maintenance of renal stem cell populations	2.39e-10	[12]
GADD45B	Pivotal for stress resilience during renal morphogenesis	1.64e-9	[13]
MSX2	Prevents osteogenic differentiation of human renal interstitial fibroblasts	2.16e-9	[14]
CEBPB	A decrease of CEBPB leads to the down-regulation of its target miRNA (miR-223, miR-21 and miR-181b), the latter inhibiting the expression of Bsnd and Ranbp3l. BSND is specific to kidney tissues, involved in making barttin, an essential subunit for chloride channels	3.18e-9	[15,16]
SAMD4A	Identifies tubule cells	4.70e-9	[17]
JUNB (AP-1)	Acts as a point of collaboration between the BMP7 and FGF9 signaling pathways—important in early nephrogenesis	6.40e-9	[2]
BACH2	A key transcription factor in the transition from self-renewal to differentiation	1.77e-8	[18]
MAFF	Plays a role in control of signaling in response to retinoids—opposes effects of MAFB (crucial for the differentiation of podocytes)	1.77e-8	[19,20]

NFATC2	Identified in proximal tubule progenitor cells	2.19e-8	[21]
CREB5	Present in a subset of renal stromal progenitors	2.95e-8	[22]
ID4	Found in developing nephron epithelium	3.01e-8	[23]
Zswim6	Some expression in tubule and collecting duct cells	3.39e-8	[24]
KLF10	Involved in vascular remodeling	7.15e-8	[25]
Herpud1	Upregulates with WNT1 signaling. WNT1 signaling is important in nephrogenesis.	2.34e-7	[26]
Hes4	Modulates NOTCH signaling in neural crest cells. Neural crest cells may be involved in kidney organogenesis by providing inductive signals and contributing cells of the renal stroma.	3.07e-7	[27,28]
EMP1	Expression corresponds to a more mature stage in ureteric bud development	4.25e-7	[29]
EXT1	Encodes glycosyltransferase required for synthesis of heparan sulfate glycosaminoglycans in the glomerular basement membrane	4.94e-7	[30]
SLIT2	Normally known for its function in axon guidance and cell migration, has recently been shown to be a signal transduced in the nephrogenic mesenchyme. Controls UB branching specificity, likely by restricting GNDF expression.	8.52e-7	[31]
CAP mesenchyme			
NRK	Involved in the induction of actin polymerization in late embryogenesis	2.94e-52	[32]
TRABD2B(tiki)	A modulator of Wnt signaling, important to early nephrogenesis	1.12e-50	[33]
DLK1	Maintains the size of the progenitor cell pool by inhibiting the formation of mature osteoblasts and adipocytes	1.10e-48	[34]
WT1	Marker of renal progenitor cell populations in the CAP mesenchyme	2.70e-41	[35]
ITAGA8	Present in metanephric mesenchyme cells during development and in mesangial cells of the glomerulus. Knockout results in the absence of kidney development. Also contributes to regulation of cell proliferation and apoptosis in renal glomerular cells.	6.18e-40	[36]
CITED1	Identified in self-renewing nephrogenic progenitor cells	6.34e-40	[37]
CTNNA2	Involved in neurite growth and migration	9.96e-35	[38]

SYT1	Known for involvement in neuronal signaling, but recently also implicated in podocytes	1.56e-26	[39]
NRG3	Specific for glomerular capillaries. Binds erb and erb4 that are limited to UB cell populations.	1.24e-25	[40,41]
Highly proliferating cells			
MKI67	Proliferation marker and cell cycle regulator	1.75e-109	[42]
TOP2A	Critical to DNA replication and cell proliferation	5.47e-100	[43]
DLGAP5	Cell cycle-related gene	1.76e-97	[44]
UBE2C	Cell cycle-related expression	9.93e-92	[45]
Mature podocyte and glomerular cells			
HIST1H3J	High expression in tubules and glomeruli	2.58e-5	
MAFB	Crucial for differentiation of progenitor cells into podocytes	3.30e-5	[46]
PLCG2	Encodes proteins that modulate actin cytoskeleton and signaling at the slit diaphragm through calcium regulation	3.62e-5	[47]
BAIAP2	Involved in podocyte migration	1.96e-2	[48]
Non-specific, involved widely in embryogenesis			
CDH12	Expressed widely in embryonic tissues. Has a high expression in human fetal kidneys. Specifically, the ureteric bud and CAP mesenchyme cell populations have a high expression level. Expression levels were also localized to proximal tubules of the developing kidney.	1.84e-8	[49]
KCNJ3	Expressed in specific nephron segments in the developing and mature kidney.	3.87e-8	[50]
HPSE2	Has been documented to have enriched expression in renal progenitor organoid cells	1.91e-7	[51]
EPHA5	Widely expressed during organogenesis	1.02e-6	[52]
PDE7B	Anti-tumorigenic effect in cancer cells	2.12e-6	[53]
TMTC1	High expression kidney tubules and glomeruli	2.67e-6	[54]
CHRM3	Expressed in renal epithelia with unknown functions	3.26e-6	[55]
GALNTL6	Involved in early embryogenesis	5.39e-6	[56]
NRXN1	Expressed in cells of metanephric mesenchyme	6.33e-6	[57]
HS3ST5	Involved in kidney morphogenesis	8.30e-6	[58]

MMP16	Involved in renal development	1.39e-5	[59]
Mir99AHG	Involved in modulation of NOTCH signaling, important in organogenesis	2.11e-5	[60]
CPED1	Involved in Wnt signaling, important to embrogenesis	2.12e-5	[61]
KCTD12	Involved in many processes in embryonic development	3.57e-5	[62]
SOX6	Important for the differentiation of renal stem cells to renin-producing JG cells	4.82e-5	[63]

Bibliography

1. Takasato M, Er PX, Chiu HS, Little MH. Generation of kidney organoids from human pluripotent stem cells. *Nat Protoc*. 09 2016;11(9):1681-92. doi:10.1038/nprot.2016.098
2. Khoshdel Rad N, Aghdami N, Moghadasali R. Cellular and Molecular Mechanisms of Kidney Development: From the Embryo to the Kidney Organoid. *Front Cell Dev Biol*. 2020;8:183. doi:10.3389/fcell.2020.00183
3. Wellik DM, Hawkes PJ, Capecchi MR. Hox11 paralogous genes are essential for metanephric kidney induction. *Genes Dev*. Jun 01 2002;16(11):1423-32. doi:10.1101/gad.993302
4. Gong KQ, Yallowitz AR, Sun H, Dressler GR, Wellik DM. A Hox-Eya-Pax complex regulates early kidney developmental gene expression. *Mol Cell Biol*. Nov 2007;27(21):7661-8. doi:10.1128/MCB.00465-07
5. Francipane MG, Han B, Lagasse E. Host Lymphotoxin-β Receptor Signaling Is Crucial for Angiogenesis of Metanephric Tissue Transplanted into Lymphoid Sites. *Am J Pathol*. Jan 2020;190(1):252-269. doi:10.1016/j.ajpath.2019.08.018
6. Couillard M, Trudel M. C-myc as a modulator of renal stem/progenitor cell population. *Dev Dyn*. Feb 2009;238(2):405-14. doi:10.1002/dvdy.21841
7. Kang HS, Beak JY, Kim YS, Herbert R, Jetten AM. Glis3 is associated with primary cilia and Wwtr1/TAZ and implicated in polycystic kidney disease. *Mol Cell Biol*. May 2009;29(10):2556-69. doi:10.1128/MCB.01620-08
8. Chung JJ, Goldstein L, Chen YJ, et al. Single-Cell Transcriptome Profiling of the Kidney Glomerulus Identifies Key Cell Types and Reactions to Injury. *J Am Soc Nephrol*. Oct 2020;31(10):2341-2354. doi:10.1681/ASN.2020020220
9. Suk FM, Chang CC, Lin RJ, et al. ZFP36L1 and ZFP36L2 inhibit cell proliferation in a cyclin D-dependent and p53-independent manner. *Sci Rep*. Feb 09 2018;8(1):2742. doi:10.1038/s41598-018-21160-z
10. Yang H, Zhu J, Guo H, et al. Molecular cloning, characterization, and functional analysis of the uncharacterized C11orf96 gene. *BMC Vet Res*. May 10 2022;18(1):170. doi:10.1186/s12917-022-03224-5
11. Nyström J, Hultenby K, Ek S, et al. CRIM1 is localized to the podocyte filtration slit diaphragm of the adult human kidney. *Nephrol Dial Transplant*. Jul 2009;24(7):2038-44. doi:10.1093/ndt/gfn743
12. Curci C, Sallustio F, Picerno A, et al. P0021 Long non-coding RNAs HOTAIR AND LINC00511 can explain human renal stem/progenitor cells capacity to repair damage induced by cisplatin. *Nephrology Dialysis Transplantation*. 2020;35(Supplement_3)doi:10.1093/ndt/gfaa142.P0021

13. Burbridge K, Holcombe J, Weavers H. Metabolically active and polyploid renal tissues rely on graded cytoprotection to drive developmental and homeostatic stress resilience. *Development*. Apr 15 2021;148(8)doi:10.1242/dev.197343
14. Cui Y, Zeng F, Zhu Z, et al. Suppression of osteogenic-like differentiation in human renal interstitial fibroblasts by miRNA-410-3p through MSX2. *Transl Androl Urol*. Oct 2020;9(5):2082-2093. doi:10.21037/tau-20-607
15. Ke P, Qian L, Zhou Y, et al. Identification of hub genes and transcription factor-miRNA-mRNA pathways in mice and human renal ischemia-reperfusion injury. *PeerJ*. 2021;9:e12375. doi:10.7717/peerj.12375
16. Estévez R, Boettger T, Stein V, et al. Barttin is a Cl- channel beta-subunit crucial for renal Cl- reabsorption and inner ear K+ secretion. *Nature*. Nov 29 2001;414(6863):558-61. doi:10.1038/35107099
17. Schena FP, Rossini M, Abbrescia DI, Zaza G. The molecular mechanisms of inflammation and scarring in the kidneys of immunoglobulin A nephropathy : Gene involvement in the mechanisms of inflammation and scarring in kidney biopsy of IgAN patients. *Semin Immunopathol*. Oct 2021;43(5):691-705. doi:10.1007/s00281-021-00891-8
18. Huang B, Liu Z, Vonk A, Zeng Z, Li Z. Epigenetic regulation of kidney progenitor cells. *Stem Cells Transl Med*. Jun 2020;9(6):655-660. doi:10.1002/sctm.19-0289
19. Burrow CR. Retinoids and renal development. *Exp Nephrol*. 2000;8(4-5):219-25. doi:10.1159/000020672
20. Tsuchiya H, Oura S. Involvement of MAFB and MAFF in Retinoid-Mediated Suppression of Hepatocellular Carcinoma Invasion. *Int J Mol Sci*. May 13 2018;19(5)doi:10.3390/ijms19051450
21. Langworthy M, Zhou B, de Caestecker M, Moeckel G, Baldwin HS. NFATc1 identifies a population of proximal tubule cell progenitors. *J Am Soc Nephrol*. Feb 2009;20(2):311-21. doi:10.1681/ASN.2008010094
22. Tanigawa S, Tanaka E, Miike K, et al. Generation of the organotypic kidney structure by integrating pluripotent stem cell-derived renal stroma. *Nat Commun*. Feb 01 2022;13(1):611. doi:10.1038/s41467-022-28226-7
23. Treacy NJ, Clerkin S, Davis JL, et al. Growth and differentiation of human induced pluripotent stem cell (hiPSC)-derived kidney organoids using fully synthetic peptide hydrogels. *Bioact Mater*. Mar 2023;21:142-156. doi:10.1016/j.bioactmat.2022.08.003
24. Karlsson M, Zhang C, Méar L, et al. A single-cell type transcriptomics map of human tissues. *Sci Adv*. Jul 2021;7(31)doi:10.1126/sciadv.abh2169
25. Nicoleau S, Fellows A, Wojciak-Stothard B. Role of Krüppel-like factors in pulmonary arterial hypertension. *Int J Biochem Cell Biol*. May 2021;134:105977. doi:10.1016/j.biocel.2021.105977
26. Chtarbova S, Nimmrich I, Erdmann S, et al. Murine Nr4a1 and Herpud1 are up-regulated by Wnt-1, but the homologous human genes are independent from beta-catenin activation. *Biochem J*. Nov 01 2002;367(Pt 3):723-8. doi:10.1042/BJ20020699
27. Huang X, Zhang L, Yang S, Zhang Y, Wu M, Chen P. Coordinate FGF and Notch Signaling to Modulate Gastrulation via Regulating Cell Fate Specification and Cell Migration in. *Genes (Basel)*. Nov 18 2020;11(11)doi:10.3390/genes11111363
28. Itäranta P, Viiri K, Kaartinen V, Vainio S. Lumbo-sacral neural crest derivatives fate mapped with the aid of Wnt-1 promoter integrate but are not essential to kidney development. *Differentiation*. Feb 2009;77(2):199-208. doi:10.1016/j.diff.2008.10.007

29. Shah MM, Tee JB, Meyer T, et al. The instructive role of metanephric mesenchyme in ureteric bud patterning, sculpting, and maturation and its potential ability to buffer ureteric bud branching defects. *Am J Physiol Renal Physiol*. Nov 2009;297(5):F1330-41. doi:10.1152/ajprenal.00125.2009
30. Roberts IS, Gleadle JM. Familial nephropathy and multiple exostoses with exostosin-1 (EXT1) gene mutation. *J Am Soc Nephrol*. Mar 2008;19(3):450-3. doi:10.1681/ASN.2007080842
31. Grieshammer U, Le Ma, Plump AS, Wang F, Tessier-Lavigne M, Martin GR. SLIT2-mediated ROBO2 signaling restricts kidney induction to a single site. *Dev Cell*. May 2004;6(5):709-17. doi:10.1016/s1534-5807(04)00108-x
32. Nakano K, Kanai-Azuma M, Kanai Y, et al. Cofilin phosphorylation and actin polymerization by NRK/NESK, a member of the germinal center kinase family. *Exp Cell Res*. Jul 15 2003;287(2):219-27. doi:10.1016/s0014-4827(03)00136-8
33. Malinauskas T, Jones EY. Extracellular modulators of Wnt signalling. *Curr Opin Struct Biol*. Dec 2014;29:77-84. doi:10.1016/j.sbi.2014.10.003
34. Dekel B, Metsuyanim S, Schmidt-Ott KM, et al. Multiple imprinted and stemness genes provide a link between normal and tumor progenitor cells of the developing human kidney. *Cancer Res*. Jun 15 2006;66(12):6040-9. doi:10.1158/0008-5472.CAN-05-4528
35. Kreidberg JA. WT1 and kidney progenitor cells. *Organogenesis*. 2010;6(2):61-70. doi:10.4161/org.6.2.11928
36. Marek I, Hilgers KF, Rascher W, Woelfle J, Hartner A. A role for the alpha-8 integrin chain (itga8) in glomerular homeostasis of the kidney. *Mol Cell Pediatr*. Oct 01 2020;7(1):13. doi:10.1186/s40348-020-00105-5
37. Murphy AJ, Pierce J, de Caestecker C, et al. SIX2 and CITED1, markers of nephronic progenitor self-renewal, remain active in primitive elements of Wilms' tumor. *J Pediatr Surg*. Jun 2012;47(6):1239-49. doi:10.1016/j.jpedsurg.2012.03.034
38. Schaffer AE, Breuss MW, Caglayan AO, et al. Biallelic loss of human CTNNA2, encoding αN-catenin, leads to ARP2/3 complex overactivity and disordered cortical neuronal migration. *Nat Genet*. Aug 2018;50(8):1093-1101. doi:10.1038/s41588-018-0166-0
39. Pattaro C, De Grandi A, Vitart V, et al. A meta-analysis of genome-wide data from five European isolates reveals an association of COL22A1, SYT1, and GABRR2 with serum creatinine level. *BMC Med Genet*. Mar 11 2010;11:41. doi:10.1186/1471-2350-11-41
40. Lake BB, Chen S, Hoshi M, et al. A single-nucleus RNA-sequencing pipeline to decipher the molecular anatomy and pathophysiology of human kidneys. *Nat Commun*. Jun 27 2019;10(1):2832. doi:10.1038/s41467-019-10861-2
41. Melenhorst WB, Mulder GM, Xi Q, et al. Epidermal growth factor receptor signaling in the kidney: key roles in physiology and disease. *Hypertension*. Dec 2008;52(6):987-93. doi:10.1161/HYPERTENSIONAHA.108.113860
42. Sun X, Kaufman PD. Ki-67: more than a proliferation marker. *Chromosoma*. Jun 2018;127(2):175-186. doi:10.1007/s00412-018-0659-8
43. Champoux JJ. DNA topoisomerases: structure, function, and mechanism. *Annu Rev Biochem*. 2001;70:369-413. doi:10.1146/annurev.biochem.70.1.369
44. Zhang H, Liu Y, Tang S, et al. Knockdown of DLGAP5 suppresses cell proliferation, induces G. *Exp Ther Med*. Nov 2021;22(5):1245. doi:10.3892/etm.2021.10680

45. Townsley FM, Aristarkhov A, Beck S, Hershko A, Ruderman JV. Dominant-negative cyclin-selective ubiquitin carrier protein E2-C/UbcH10 blocks cells in metaphase. *Proc Natl Acad Sci U S A*. Mar 18 1997;94(6):2362-7. doi:10.1073/pnas.94.6.2362
46. Brunskill EW, Georgas K, Rumballe B, Little MH, Potter SS. Defining the molecular character of the developing and adult kidney podocyte. *PLoS One*. 2011;6(9):e24640. doi:10.1371/journal.pone.0024640
47. Lane BM, Cason R, Esezobor CI, Gbadegesin RA. Genetics of Childhood Steroid Sensitive Nephrotic Syndrome: An Update. *Front Pediatr*. 2019;7:8. doi:10.3389/fped.2019.00008
48. Yanagida-Asanuma E, Asanuma K, Kim K, et al. Synaptopodin protects against proteinuria by disrupting Cdc42:IRSp53:Mena signaling complexes in kidney podocytes. *Am J Pathol*. Aug 2007;171(2):415-27. doi:10.2353/ajpath.2007.070075
49. van der Zanden LFM, van Rooij IALM, Quaedackers JSLT, et al. as a Candidate Gene for Kidney Injury in Posterior Urethral Valve Cases: A Genome-wide Association Study Among Patients with Obstructive Uropathies. *Eur Urol Open Sci*. Jun 2021;28:26-35. doi:10.1016/j.euros.2021.04.001
50. Surendran K, Kopan R. Chromatin-based mechanisms of renal epithelial differentiation. *J Am Soc Nephrol*. Jul 2011;22(7):1208-12. doi:10.1681/ASN.2010101018
51. Subramanian A, Sidhom EH, Emani M, et al. Single cell census of human kidney organoids shows reproducibility and diminished off-target cells after transplantation. *Nat Commun*. Nov 29 2019;10(1):5462. doi:10.1038/s41467-019-13382-0
52. Park JE, Son AI, Zhou R. Roles of EphA2 in Development and Disease. *Genes (Basel)*. Jul 01 2013;4(3):334-57. doi:10.3390/genes4030334
53. Sun Y, Zou J, Ouyang W, Chen K. Identification of. *Cancer Manag Res*. 2020;12:5701-5712. doi:10.2147/CMAR.S259192
54. Uhlén M, Fagerberg L, Hallström BM, et al. Proteomics. Tissue-based map of the human proteome. *Science*. Jan 23 2015;347(6220):1260419. doi:10.1126/science.1260419
55. Jain S, Chen F. Developmental pathology of congenital kidney and urinary tract anomalies. *Clin Kidney J*. Jun 2019;12(3):382-399. doi:10.1093/ckj/sfy112
56. Nairn AV, Aoki K, dela Rosa M, et al. Regulation of glycan structures in murine embryonic stem cells: combined transcript profiling of glycan-related genes and glycan structural analysis. *J Biol Chem*. Nov 02 2012;287(45):37835-56. doi:10.1074/jbc.M112.405233
57. Potter SS, Brunskill EW, Patterson LT. Microdissection of the gene expression codes driving nephrogenesis. *Organogenesis*. 2010;6(4):263-9. doi:10.4161/org.6.4.12682
58. Patel VN, Lombaert IM, Cowherd SN, et al. Hs3st3-modified heparan sulfate controls KIT+ progenitor expansion by regulating 3-O-sulfotransferases. *Dev Cell*. Jun 23 2014;29(6):662-73. doi:10.1016/j.devcel.2014.04.024
59. Lenz O, Elliot SJ, Stetler-Stevenson WG. Matrix metalloproteinases in renal development and disease. *J Am Soc Nephrol*. Mar 2000;11(3):574-581. doi:10.1681/ASN.V113574
60. Xu J, Xu W, Yang X, Liu Z, Zhao Y, Sun Q. LncRNA MIR99AHG mediated by FOXA1 modulates NOTCH2/Notch signaling pathway to accelerate pancreatic cancer through sponging miR-3129-5p and recruiting ELAVL1. *Cancer Cell Int*. Dec 15 2021;21(1):674. doi:10.1186/s12935-021-02189-z
61. Bokhari HA, Shaik NA, Banaganapalli B, et al. Whole exome sequencing of a Saudi family and systems biology analysis identifies. *Saudi J Biol Sci*. Jun 2020;27(6):1494-1502. doi:10.1016/j.sjbs.2020.04.011

62. Abbaszadegan MR, Taghehchian N, Li L, Aarabi A, Moghbeli M. Contribution of KCTD12 to esophageal squamous cell carcinoma. *BMC Cancer*. Aug 29 2018;18(1):853. doi:10.1186/s12885-018-4765-z
63. Saleem M, Hodgkinson CP, Xiao L, et al. Sox6 as a new modulator of renin expression in the kidney. *Am J Physiol Renal Physiol*. Feb 01 2020;318(2):F285-F297. doi:10.1152/ajprenal.00095.2019
